# Modeling human neurodegeneration with iPSCs: opportunities, limitations, and the challenge of aging

**DOI:** 10.1172/JCI209602

**Published:** 2026-10-15

**Authors:** Shijie Wang, Lisa Elia, Weiyi Liu, Steven Finkbeiner

**Affiliations:** 1 Center for Systems and Therapeutics, Gladstone Institutes, San Francisco, California, USA.; 2 Gladstone Institutes, San Francisco, California, USA.; 3 Department of Metabolic Biology and Nutrition, University of California, Berkeley, Berkeley, CA, USA.; 4 Molecular Toxicology Graduate Program, UCB, Berkeley, California, USA.; 5 Department of Neurology,; 6 Department of Physiology, and; 7 Neuroscience Graduate Program and Biomedical Sciences Program, UCSF, San Francisco, California, USA.

## Abstract

Most neurodegenerative diseases reflect patient-specific combinations of polygenic risk variants interacting with aging and environmental factors, rather than a single causative mutation. Phase III clinical trial failure rates for neurodegenerative indications exceed 95%, reflecting in part the limitations of existing preclinical systems to fully model the human genetic context of disease. Patient-derived induced pluripotent stem cells (iPSCs) have the potential to address this shortcoming by faithfully recapitulating human cell biology and preserving each patient’s complete genetic architecture. This Review examines iPSC-based models of neurodegeneration across three themes. First, we discuss mechanisms revealed by these models, from convergent pathways uncovered by familial mutations to early evidence that sporadic disease biology is accessible at sufficient cohort scale. Second, we evaluate culture systems from 2-dimensional monocultures to organoids, examining how increasing complexity expands addressable biological questions while introducing trade-offs. Third, we address the aging problem: reprogramming erases epigenetic signatures of donor aging, removing the cellular context in which late-onset sporadic neurodegeneration emerges. We discuss strategies approximating aging in iPSC-derived neurons and highlight direct conversion strategies that preserve native epigenetic age, enabling access to age-dependent phenotypes. We propose a framework matching model systems to biological questions, improving mechanistic understanding and therapeutic development.

## Introduction

As the global burden of neurodegenerative diseases (NDDs) grows with aging populations, developing effective treatments has become an urgent priority. Despite decades of research, phase III clinical trial failure rates for NDDs exceed 95%, and only 3% to 5% of preclinical findings have progressed to regulatory approval (1, 2). This poor record of translation reflects, in part, the limited predictive power of existing preclinical model systems and underscores the need to develop human-relevant models that more faithfully capture the biology driving human neurodegeneration.

Modeling NDDs is challenging for several reasons. First, most cases of Alzheimer disease (AD), Parkinson disease (PD), Lewy body dementia (LBD), amyotrophic lateral sclerosis (ALS), and frontotemporal dementia (FTD) are sporadic, arising not from a single causative mutation but from the cumulative interaction of polygenic risk variants, each of modest individual effect, combined with stochastic and environmental factors (Figure 1). These cannot be faithfully recapitulated in nonhuman models, because they are unknown, it is infeasible, or both. Second, clinical presentation does not map reliably onto molecular etiology. For instance, mutations in dozens of distinct genes can produce clinically indistinguishable ALS syndromes (3), whereas a single variant, *C9orf72* repeat expansion, can lead to either ALS or FTD (4, 5), a mechanistically unexplained divergence that challenges single-gene modeling approaches. Third, aging is the primary risk factor for all major NDDs, but it is a complex, progressive, multisystem process that remains incompletely understood at the mechanistic level. Moreover, it is unclear how aging trajectories in short-lived organisms compare with the decades-long aging process in humans.

The discovery that adult somatic cells can be reprogrammed to a pluripotent state by defined transcription factors (6, 7) created a means to address the first two challenges. Patient-derived induced pluripotent stem cells (iPSCs) preserve the full genetic background of the donor, including the polygenic variants that underlie sporadic risk, while providing renewable, expandable sources of disease-relevant human neurons and glia (8). Yet iPSCs carry a fundamental biological limitation: reprogramming erases the epigenetic signatures of aging accumulated in the donor’s cells, resetting neurons to a fetal-like identity. For diseases whose onset depends on decades of accumulated molecular change, this epigenetic reset removes the very context in which neurodegeneration emerges.

This Review examines the current state and limitations of iPSC-based models of NDDs across three connected themes: (a) what current iPSC models have established about disease mechanisms, including convergent and disease-specific pathways affected by high-penetrance familial mutations, and early evidence that sporadic disease can be modeled with iPSCs; (b) the range of culture systems available and the practical considerations in model system selection; and (c) strategies that approximate aging in iPSC-derived neurons or preserve the donor’s biological age via transdifferentiation. Throughout, we provide a practical framework for matching model systems to biological questions, with the goal of improving how iPSC-based approaches contribute to mechanistic understanding and therapeutic development.

## What iPSC models have taught us

### The promise of patient-derived cell models.

An NDD model must satisfy three requirements. First, it must recapitulate human biology, as many disease-relevant molecular features are human specific. For instance, the TDP-43 splicing targets most relevant to ALS neurodegeneration, including the intronic sequences containing the TDP-43 binding sites in *UNC13A* and *STMN2*, are poorly conserved across species (9, 10). Although the genes themselves are present in rodents, the corresponding splicing regulation does not occur in rodent models. Similarly, genomic regions encoding AD risk variants have undergone accelerated evolution in the human lineage, with no rodent equivalent (11).

Second, the model must preserve the patient’s full genetic architecture. Rare, high-penetrance familial mutations in *APP*, *PSEN1*, and *PSEN2* (AD); *SNCA*, *LRRK2*, and *PRKN* (PD); and *TARDBP*, *SOD1*, and *C9orf72* (ALS-FTD) act as primary disease drivers, but they account for fewer than 5% to 15% of cases across most conditions (12). Still, the overall heritability of NDD risk, the proportion of disease risk attributable to genetic rather than environmental variation, is substantial: approximately 70% to 80% for AD (largely driven by *APOE4*), 40% to 60% for ALS, and roughly 30% for PD (13–15). Whole-genome and whole-exome sequencing also show that rare variants may contribute more to risk of disease than once thought, accounting for over a quarter of apparently sporadic ALS (16). Genome-wide association studies (GWAS) have revealed hundreds of common variants that each confer modest individual risk (17, 18). The portion of heritability not explained by known high-penetrance mutations reflects a polygenic architecture in which many variants of individually modest effect combine to confer disease susceptibility. This architecture is impossible to re-create through genetic engineering when the causal variants are not fully known.

Patient-derived iPSCs address both of these requirements simultaneously (6, 7). They maintain the donor’s complete genomic landscape, whether a single familial mutation or a constellation of common and rare variants underlying polygenic sporadic risk, while providing renewable sources of disease-relevant human cell types that are otherwise inaccessible from living patients (8, 19). A complementary strategy involves engineering defined mutations onto a common background, such as the KOLF2.1J reference line (20), reducing interline variance. However, these engineered lines carry no accompanying clinical data, making it unclear, in some cases, if a person with that genetic background and engineered mutation would have developed disease. Thus, their uncertain value for predictive, patient-anchored modeling brings a trade-off that should be weighed against patient-derived approaches.

Third, a model must exhibit measurable disease-relevant phenotypes, whether molecular, such as transcriptomic changes, or macroscopic, such as neuronal death. Whether a given iPSC-derived model also produces a measurable phenotype, however, is not guaranteed: it depends on the disease, the cell types generated, and how well the model captures the aging context in which neurodegeneration unfolds, the central challenge to which we return throughout this Review.

### From genetics to cellular phenotype.

Genetic variants do not directly determine cellular phenotypes. Instead, they perturb hierarchical networks of biological pathways spanning multiple scales (Figure 1). Individual nucleotide changes directly alter protein structure or stability, or act indirectly, through effects on transcription factor binding sites, splicing regulatory sequences, or chromatin configuration to alter protein levels, which in turn affect protein-protein interactions and signaling cascades, eventually compromising the cellular processes whose failure contributes to disease initiation (21). Most GWAS hits identify a locus that is in linkage disequilibrium with a variant that causes the association rather than being the causal variant itself. GWAS loci typically reside in noncoding regions, and fine-mapping efforts show that the causal variant often resides in a noncoding regulatory region rather than in an exon, a pattern confirmed by functional genomics analyses of NDD loci (3).

Not all observable changes in iPSC-derived neurons are pathogenic. Some are incidental consequences of upstream perturbations; others are adaptive responses that initially protect the cell. For instance, the formation of inclusion bodies in neurons expressing mutant huntingtin, the causative protein in Huntington disease (HD), reduces levels of diffuse toxic protein and decreases cell death risk, functioning as a coping response rather than a driver of pathology (22). Distinguishing pathogenic from adaptive or incidental phenotypes is one of the central challenges of this field.

The optimal assay window shifts with disease architecture. Common risk variants identified by GWAS typically have a small effect size, and the functional signal proximal to any of these variants will be small, often below the detection threshold of standard assays: the common variants associated with late-onset AD typically confer approximately 10% risk or protection (23), effect sizes close to the noise floor of iPSC model systems. Therefore, for polygenic disease, phenotypic assays positioned at convergent downstream nodes, where effects of multiple upstream variants accumulate or synergize, may be better suited to detect a meaningful signal and are less sensitive to technical variability between individual lines. For high-penetrance monogenic mutations, the relationship is reversed: a single variant is sufficient to drive disease with a large effect size, and the clearest signal is often found closest to the primary molecular event, as homeostatic mechanisms progressively buffer the impact of the mutation with each step downstream (Figure 1).

### High-penetrance mutations reveal convergent and disease-specific phenotypes.

iPSC-derived models have successfully recapitulated multiple hallmark features of NDDs (19, 24, 25). In particular, patient-derived neurons carrying high-penetrance familial mutations have revealed both disease-specific cellular vulnerabilities and convergent mechanisms across otherwise distinct conditions (Figure 2).

Across AD, PD, LBD, ALS, and FTD, a striking number of pathological mechanisms emerge as shared themes. Mitochondrial dysfunction, autophagy deficits and lysosomal impairment, ER stress, oxidative stress, and axonal transport deficits appear in iPSC-derived neurons from all these conditions (Supplemental Tables 1–4, and Figure 2; supplemental material available online with this article; https://doi.org/10.1172/JCI209602DS1), suggesting that convergent failure of core cellular quality control pathways is a shared rather than a disease-specific feature of neurodegeneration, one that may reflect core nodes of vulnerability engaged by any stressed neuron (26). Neuroinflammatory signatures likewise appear across conditions in the form of astrocyte and microglial dysfunction (27, 28), demonstrating that generating glial cell types alongside neurons is a scientific necessity for understanding how human neurons degenerate.

Beyond these convergent features, each disease also exhibits phenotypes that are distinct. AD models carrying *APP* duplications, *PSEN1* variants, or Down syndrome (trisomy 21) reliably recapitulate elevated amyloid secretion, tau hyperphosphorylation, and synaptic dysfunction (24, 29, 30) (Figure 2 and Supplemental Table 1). PD and LBD models carrying *SNCA* triplication, *LRRK2* mutations, or *GBA* mutations reproduce α-synuclein accumulation, impaired mitophagy, and lysosomal defects (31–33) (Figure 2 and Supplemental Table 2). ALS and FTD neurons harboring hexanucleotide expansions in *C9orf72*, *TDP-43* variants, and *SOD1* variants recapitulate RNA foci and nucleocytoplasmic transport failure (34–36) (Figure 2 and Supplemental Tables 3 and 4). Axonal transport defects have been reported in *TDP-43* mutant lines (37). However, such axonal phenotypes are not seen consistently across lines and protocols, and negative results are likely underreported, so they should be interpreted with caution.

The recurrence of the same core cellular pathways in neurons carrying mechanistically unrelated high-penetrance mutations supports the inference that polygenic risk variants for sporadic disease act through the same convergent nodes. A given patient, however, is unlikely to harbor risk variants that impair all of these pathways equally. The particular combination of variants a patient carries will tend to favor some pathways over others, which motivates the concept of a pathway-specific polygenic risk score to guide both phenotypic assay selection and patient stratification (38).

### From familial mutations to sporadic disease.

The vast majority of iPSC models mentioned above were built on familial mutations, which are tractable precisely because their penetrance is high and phenotypes relatively robust, but which account for fewer than 1 in 10 patients with AD, PD, or ALS. Fewer than 10% of published studies have attempted to model sporadic disease using lines from patients without a known causal mutation, and where sporadic lines were included, phenotypes were generally more modest and variable than in familial counterparts (24, 39) (Figure 2).

The first iPSC-derived AD study included two sporadic AD lines, only one of which recapitulated hallmarks seen in familial lines, suggesting that sporadic biology overlaps with familial biology but requires specific conditions to emerge (24). A subsequent study deriving cortical neurons from 53 aged, cognitively diverse donors demonstrated that neuronal network activity reflects donor neuropathology independent of genotype (25), providing evidence that sporadic AD phenotypes are detectable in iPSC-derived cells when cohorts are large enough. The most sustained effort to model sporadic disease at scale has come from the ALS field (40–44). The Answer ALS consortium has assembled over 1,100 patient and control iPSC lines, including approximately 100 lines from patients with sporadic disease, and has begun to reveal transcriptomic heterogeneity that is invisible in small familial cohorts (40, 41). That heterogeneity may reflect biologically distinct patient subgroups whose identification is a prerequisite for any serious precision medicine effort. In an independent cohort, large-scale screening of iPSC-derived motor neurons from 100 sporadic ALS patients showed reduced survival and accelerated neurite degeneration relative to controls (45), among the clearest sporadic patient–versus-control differences reported to date, in contrast with earlier cohorts, where differences were modest (40).

The clearest demonstration that iPSC models can capture genetic risk comes from studies of *APOE4* in isogenic lines. *APOE4* is the strongest common risk driver for late-onset AD, present in approximately 40% of AD cases despite being carried by only 15% of the general population (46). Isogenic conversion of *APOE3* to *APOE4* in iPSC-derived models recapitulated the cholesterol metabolism defects, high amyloid secretion, and microglial dysfunction characteristic of AD (47–49). This isogenic design succeeded because *APOE4* carries an unusually large effect size for a common variant. As the field moves toward investigating true polygenic risk, where no single variant approaches the effect size of *APOE4*, isogenic correction alone is insufficient. Detecting the combined effects of many small-effect variants will require large, carefully phenotyped cohorts and analytical frameworks capable of resolving biologically meaningful subgroups within sporadic populations.

Familial and sporadic disease are not fundamentally different entities. Virtually every gene that causes dominant neurodegeneration through rare, high-penetrance mutations also harbors weaker common variants that confer sporadic disease risk (12). For example, *LRRK2(G2019S)* increases PD risk approximately 20-fold, while the common *LRRK2(G2385R)* variant confers a more modest 2-fold increase (50, 51). Furthermore, the familial–sporadic boundary can blur in practice: more individuals carrying *C9orf72* repeat expansions lack a family history of ALS than have one (52). More broadly, sporadic denotes the absence of an apparent family history rather than the absence of disease risk. What familial iPSC models have therefore provided is a foundation: the pathways they illuminate are likely the same pathways that, when more subtly perturbed by combinations of common variants, drive the majority of human neurodegeneration, often with the help of environmental factors. The question is no longer whether iPSC models can capture the biology of sporadic disease but whether the field will commit to the scale and analytical rigor that capturing it requires.

## From 2D monocultures to 3D organoids

Having established what iPSC models can reveal about disease genetics, this section turns to how to build such models. The choice of culture system, from 2D monocultures to multicellular cocultures to 3D organoids, is not a matter of technical preference but a scientific decision that should be driven by the biological question at hand.

### Matching cell type to question.

Patient-derived iPSCs can now be differentiated into the neuronal subtypes affected in specific diseases: cortical excitatory and inhibitory neurons for AD and FTD (53–58), midbrain dopaminergic neurons for PD (59, 60), and spinal motor neurons for ALS (61, 62), with protocols also available for upper cortical motor neurons affected in ALS (63). Distinct differentiation protocols generate astrocytes (64, 65), oligodendrocytes (66), and microglia, the last of which requires a non-neural myeloid differentiation route reflecting the unique yolk sac developmental origin of these cells (67–70).

These protocols vary substantially in technical difficulty, yield, and time. Cortical excitatory neuron protocols are the fastest and most widely established. Protocols that direct differentiation into disease-relevant subtypes, such as midbrain dopaminergic or spinal motor neurons, require longer and more complex steps but have become increasingly well established and robust. Microglia differentiation remains technically demanding, and achieving high purity while maintaining a homeostatic transcriptional state in vitro is an active area of development. Transcription factor–based strategies offer faster routes: inducible expression of defined transcription factors yields microglia-like cells within 1 to 2 weeks (71, 72), and the same principle underlies rapid NGN2-based induced-neuron protocols (53, 54). These approaches are inexpensive and scalable, making them well suited for screening, but they come at the expense of the cell type–specific identity that is central to many diseases and potential confounding effects of transcription factor overexpression on disease phenotypes.

A challenge shared across all protocols is achieving functional maturation comparable to adult counterparts in vivo. iPSC-derived neurons and glia broadly retain a fetal transcriptional signature that cannot be resolved by extending culture time in 2D alone (65), and this maturation limitation shapes interpretation across all culture systems.

### From monocultures to cocultures.

Two-dimensional monoculture systems offer precise control of cellular composition, compatibility with high-throughput screening, and straightforward application of functional assays. The cell-type–specific phenotypes described above were largely established in these systems. Their limitation is that interactions among neurons, astrocytes, microglia, oligodendrocytes, and vascular cells cannot be recapitulated in single–cell-type cultures.

Coculture systems address this by combining multiple iPSC-derived cell types. ALS models incorporating patient-derived motor neurons with astrocytes carrying SOD1 or TDP-43 mutations have demonstrated non-neuron-autonomous toxicity, where mutant astrocytes accelerate motor neuron death (73–75). In AD models, neuron-astrocyte cocultures reveal astrogliosis-like phenotypes and tripartite synaptic structures absent in monocultures (76). Adding microglia enables investigation of the neuro-immune interactions characteristic of neurodegeneration (77, 78). Most coculture systems also lack myelinating oligodendrocytes, though whether this is a limitation depends on the stage being modeled. Because the early human brain contains little myelin, unmyelinated cultures may reasonably approximate early developmental stages; for adult-onset diseases in which oligodendrocyte and myelin dysfunction are increasingly implicated, however, their absence remains an important limitation. This is itself an active area of iPSC modeling, and iPSC-derived oligodendrocytes can be cocultured with neurons using established protocols (66, 79).

Nevertheless, coculture systems do not resolve the maturation limitation, and the added cellular complexity introduces new sources of variability that require adequately powered experiments to distinguish disease-relevant signals from technical noise.

### Advantages and limitations of complex organoids.

Three-dimensional organoid systems self-organize into tissue-like structures with spatial architecture, neuronal layering, and circuit connectivity not achievable in 2D cultures (80). The 3D format substantially advances the maturation trajectory: iPSC-derived astrocytes retain a fetal transcriptional signature regardless of differentiation method in 2D (65), but in cerebral organoids, mature homeostatic and synaptic programs accumulate over months (81).

This maturation advantage has direct relevance to disease modeling. The adult shift in tau splicing isoforms from 3R to 4R, which is directly relevant to AD and 4R tauopathies, does not occur in 2D iPSC-derived neurons cultured for up to 365 days but is detected in 3D human cortical organoids by 6 months (82, 83). Similarly, spontaneous TDP-43 pathology was observed in 3D cocultures of neurons and glial cells with GRN loss but not in equivalent 2D systems below 100 days of culture (84, 85). These examples establish that certain disease-relevant cellular states may be achievable in 3D systems only.

These maturation benefits, however, have a ceiling. Organoids plateau at fetal-to-early postnatal transcriptional states across all methods (86–88), and even cultures maintained for nearly 700 days correspond by epigenetic clock to early postnatal, not adult, brain (86). More complex models are also not inherently better in all respects. Organoids lack vascular perfusion, leading to necrotic or hypoxic cores that limit their size and create artificial gradients (89); they exhibit substantial batch-to-batch variability in cell type composition; and they present increased challenges for high-throughput and quantitative assays. Assembloid approaches address some of these limitations by fusing region-specific organoids and adding vascular networks to enhance maturation (40, 90), or incorporating microglia for neuro-immune studies (91), each expanding what can be asked at the cost of further technical complexity.

Another dimension of disease that is difficult to capture in a dish is that disorders such as ALS involve network-level degeneration, in which the equilibrium of vast numbers of neuronal connections progressively breaks down across distributed circuits, and they unfold through a series of pathological events whose sequence is neither fully understood nor easily recapitulated experimentally. Cortico-motor assembloids that connect cortical projection neurons to spinal motor neurons and muscle partially reconstruct the affected circuit (92). Although in vitro models can dissect specific cellular relationships within these processes, they cannot reconstruct the intact neural system.

A complementary in vivo strategy transplants human iPSC-derived organoids into the rodent CNS. Xenografted organoids become vascularized, integrate with host circuitry, and access systemic and blood-brain barrier–dependent cues difficult to reproduce in vitro, advancing maturation beyond culture (93, 94). As the methodology matures, they could become an increasingly useful hybrid platform for environment-dependent modeling, though they accelerate maturation rather than aging and develop within a xenogeneic, typically immunodeficient host.

### Practical considerations when choosing a culture system.

No single system is universally best. In general, variability and complexity are negatively correlated, so the simplest system that can model the biology necessary to address the question at hand will typically provide the most sensitive, reproducible, and statistically tractable experiments. For questions about early molecular events, pathway-level readouts intrinsic to a specific neuronal type, or high-throughput screening, 2D monocultures remain appropriate. For questions about glial contributions, or the role of neuroinflammation, incorporating relevant glial cell types is a scientific necessity rather than an optional refinement. For phenotypes that require tissue-level spatial organization or circuit dynamics, organoids and assembloids offer capabilities 2D systems cannot match. Each step up in complexity demands an explicit account of what specific biological property the added complexity enables, to justify the associated trade-offs in variability, throughput, and cost.

## Addressing the aging problem in iPSC models

### Epigenetic reset limits sporadic disease modeling.

The incidence of sporadic NDDs rises steeply with advancing age: AD risk roughly doubles every 5 years after age 65 (95), and the incidence of most NDDs increases by an order of magnitude between the sixth and ninth decades of life (26). Across all major NDDs, 85% to 95% of cases are sporadic, arising from the cumulative interaction of polygenic risk architecture, stochastic cellular events, and environmental exposures that unfold against the backdrop of aging (12, 26). Aging is therefore not merely a permissive background for neurodegeneration; it is also coupled to disease initiation and progression (96). However, the epigenetic reset that makes iPSCs valuable for regenerative medicine, including restoration of telomere length, reset of DNA methylation–based aging clocks to near-embryonic levels, and reversion to a fetal-like transcriptional state, simultaneously removes the cellular context in which late-onset neurodegeneration emerges (97). Several strategies have been developed to approximate aspects of the aged cellular state in iPSC-derived neurons. Each has demonstrated partial utility, but none recapitulates the full complexity of physiological aging.

### Genetic perturbations that mimic features of cellular aging.

Expression of progerin, a truncated lamin A splice variant that causes premature aging in Hutchinson-Gilford progeria syndrome (HGPS), accelerates multiple aging hallmarks, including nuclear abnormalities, DNA damage, and cellular senescence (98, 99). In iPSC-derived dopaminergic neurons, overexpression of progerin induced neuronal stress phenotypes, including mitochondrial dysfunction and α-synuclein inclusions reminiscent of late-onset PD (100, 101). This demonstrated that a cellular stress perturbation can lower the threshold for expression of latent genetic vulnerabilities in iPSC-derived neurons. However, patients with HGPS develop profound premature systemic aging in childhood yet maintain relatively normal cognitive function and do not develop neurodegeneration (102); the brain is spared in HGPS because progerin is not expressed there (98); and different tissues age at different rates (103, 104). Progerin overexpression is therefore best understood as a sensitization tool that reveals whether a given genetic background carries vulnerability to stress, not as a faithful model of how aging drives neurodegeneration.

Telomere shortening is among the best-supported hallmarks of aging, and evidence from telomerase-null mice show that progressive telomere attrition can drive aging-like deterioration across tissues (96, 105). Whether this process meaningfully drives aging in postmitotic neurons remains unresolved (106, 107). Telomere manipulation in iPSC-derived neurons has produced stress phenotypes overlapping with features of neuronal aging and revealed late-onset disease features (108), but like progerin, this approach captures one component of aging while leaving the broader epigenetic and transcriptomic aging program unaddressed.

Small molecule compounds that induce cellular senescence represent a third approach. The neddylation inhibitor MLN4924 induces senescence hallmarks, including cell cycle arrest and nuclear lamina disruption. In iPSC-derived neurons, it has selectively unmasked disease phenotypes in familial AD and PD models compared with isogenic controls (109, 110). Chemical “cocktails” to induce key senescence activities in stem cell–derived neurons have also unmasked phenotypes such as TDP-43 mislocalization (111).

However, the relationship between aging and senescence is complex and incompletely resolved (112–114). Cellular senescence comprises diverse, stress-induced shifts in cell state, driven largely by aging-associated stress, that can influence aging but are not synonymous with it (115, 116). Senescence states have been described in postmitotic cells such as neurons, but what senescence means in that context remains unsettled (117, 118). Aged neurons have additionally undergone decades of epigenetic changes, proteostasis deterioration, and metabolic changes that no acute pharmacological trigger reproduces.

### Environmental and chemical approaches to approximating aging.

A distinct category of approaches exposes iPSC-derived neurons and organoids to stressors and inflammatory signals characteristic of the aged brain environment, such as mitochondrial complex I inhibitors, autophagy-lysosomal pathway blockers, or serum from aged donors. Brain organoids exposed to aged donor serum developed amyloid plaque-like aggregates, phosphorylated tau, and synaptic network dysfunction reminiscent of sporadic AD (29, 119), demonstrating that non-cell-autonomous signals in aged blood can drive AD-like pathology even without familial mutations. These approaches model conditions that co-occur with aging rather than inducing the aging process itself. They are useful for examining how specific aging-associated stresses interact with a given genetic background, but they cannot address how the global epigenetic changes that accumulate over decades, keeping time, shape neurodegeneration risk. A further caveat is interpretive: perturbing pathways such as mitochondrial function, inflammation, or lysosomal activity to approximate aging can bias interpretation toward a causal role for them, when the same changes may be consequences rather than causes; this conflation could contribute to failed therapeutic programs.

### Preserving aging: transdifferentiation as the direct approach.

Aging is best understood not as a single pathway perturbation but as a gradual, system-wide decline in resilience across multiple homeostatic systems, including proteostasis, mitochondrial quality control, DNA damage response, epigenetic regulation, and immune surveillance (96, 120). No single genetic or pharmacological perturbation recapitulates this complexity.

Direct conversion, also termed transdifferentiation, transforms somatic cells, most commonly dermal fibroblasts, directly into neurons without passing through a pluripotent state. Because the cells bypass pluripotency entirely, the donor’s epigenetic age, telomere length, oxidative stress burden, and DNA damage profiles are preserved (121) — the resulting neurons carry the biological age of the person they came from. A key molecular measure of this preserved aging is the epigenetic clock, DNA methylation patterns that predict chronological age with remarkable accuracy. Whether these marks mechanistically drive aging or only correlate with it has not been directly established. The reprogramming experiment itself, however, provides relevant evidence for a causal role: erasing these marks during reprogramming to pluripotency restores youthful functional profiles, suggesting that epigenetic age marks are active contributors to the aged cellular state rather than passive signatures. Approaches that preserve endogenous epigenetic age are therefore likely more faithful representations of late-onset disease biology than methods that artificially reimpose selected aging correlates (97).

Several direct conversion strategies have been reported (Supplemental Table 5). The most extensively validated approach delivers two brain-enriched microRNAs, miR-9/9* and miR-124, to suppress fibroblast gene expression programs and drive neuronal chromatin remodeling (122). Coexpression of lineage-specific transcription factors then directs neurons toward disease-relevant subtypes, including striatal medium spiny neurons (CTIP2, DLX1, DLX2, MYT1L), motor neurons (ISL1, LHX3, NGN2), and cortical neurons (MYT1L, NEUROD2) (122–124). Transcription factor–based approaches without microRNAs provide alternatives, including ASCL1, BRN2, MYT1L, and NEUROD1 for cortical neurons (125, 126) and ASCL1, NURR1, and LMX1A for dopaminergic neurons relevant to PD (127).

The value of preserving aging is demonstrated most clearly by disease phenotypes that iPSC-derived models cannot reproduce (Supplemental Table 5). In HD, medium spiny neurons converted directly from patient fibroblasts exhibited mutant huntingtin (HTT) aggregates and spontaneous neurodegeneration; these phenotypes were absent from iPSC-derived neurons from the same patients (128, 129). Direct conversion preserves the donor’s decline in proteostasis capacity, which is required for mutant HTT to aggregate and cause cell death. Tauopathies including certain forms of FTD are defined by disruption of the 3R:4R ratio, and thus models that reflect the true endogenous isoform balance of the donor are essential for studying disease pathogenesis. Directly converted cortical neurons from patients with tauopathy express both 3R and 4R tau isoforms at their endogenous adult ratio (130), whereas iPSC-derived neurons express exclusively fetal 3R tau and do not produce 4R tau without genetic manipulation or very prolonged culture (130, 131). In AD, directly converted neurons from patient fibroblasts showed specific deficits in proteostasis and lysosomal quality control that permitted the accumulation of toxic protein aggregates and drove neurodegeneration (132). These phenotypes were absent in iPSC-derived neurons from the same patients (132, 133), consistent with the interpretation that the proteostatic decline captured by direct conversion requires the preserved aging context of the donor.

### The practical limitations of transdifferentiation.

Direct conversion has practical limitations that constrain its current use. Conversion efficiency is low and variable across protocols and cell lines: most approaches require multiple viral vectors to deliver reprogramming factors (124–126), and the limited number of neurons obtainable from primary fibroblast cultures makes large-scale experiments difficult. The resulting cultures are often heterogeneous in maturation state (126, 134). Primary fibroblasts have a finite replicative life span, limiting the ability to maintain and expand sufficient material for repeated experiments, and the use of viral integration vectors limits compatibility with downstream genetic screening approaches. Direct conversion is also practically incompatible with CRISPR-based isogenic editing. Confirming a genomic edit requires clonal expansion over many cell divisions, which is very difficult in primary fibroblasts, particularly those from older donors, whose finite replicative life span limits expansion before senescence. This makes it impractical to generate gene-corrected isogenic controls or stable CRISPR inhibition or CRISPR activation lines, leaving investigators dependent on fibroblasts from different donors and reintroducing the interindividual genetic variation that isogenic approaches are designed to eliminate. Approaches to overcome this constraint have been explored. Ectopic, excisable human telomerase reverse transcriptase (hTERT) expression may offer one partial route to extend fibroblast life span (135). However, TERT also has nonenzymatic functions beyond telomere maintenance, and its ectopic effects are incompletely understood, making it not a clean solution.

Sporadic disease modeling adds a further burden: because the aging state differs across donors, separating disease-specific signals from interindividual age variation requires cohorts with adequate donor diversity (132, 133), a requirement that is difficult to meet given current yield constraints.

These limitations do not diminish the scientific value of direct conversion but clarify where it is most appropriately applied. The specific advantage of direct conversion is access to an aging context that iPSC-derived models cannot achieve. The phenotypes that depend on this context, including tau isoform balance, age-dependent proteostasis failure, and protein aggregation, demonstrate that preserved aging is critical to model certain aspects of late-onset neurodegeneration, not only a theoretical advantage. There is also an underappreciated translational dimension. Compounds identified in iPSC-based screens that reflect a fetal epigenetic state may not retain activity in the aged neuronal environment of the patients who would receive them. A model that preserves authentic donor aging would improve identification of compounds effective in aged neurons.

*When is aging intervention necessary?* For high-penetrance familial mutations, standard iPSC-derived neurons often produce robust phenotypes without modification. For example, neurons carrying *APP* or *PSEN1* mutations show altered amyloid processing under standard conditions (24). In these settings, approximating aging is not required to detect the primary pathogenic mechanism, though it may amplify phenotypes of interest.

Other phenotypes are absent in standard iPSC neurons but can be revealed when cellular resilience is reduced. Progerin overexpression, telomere manipulation, or pharmacological senescence induction has revealed disease phenotypes in familial AD and PD neurons that were indistinguishable from isogenic controls without sensitization (101, 109). These approaches are useful for questions in which the genetic vulnerability is latent in young neurons and requires reduced cellular resilience to become expressed.

A further category of phenotypes cannot be accessed through partial aging approaches at all. The adult 3R:4R tau isoform ratio (130, 131), sporadic AD–associated proteostasis deficits (132, 133), and protein aggregation in HD and AD (128, 129) are present in directly converted neurons but absent in iPSC-derived neurons from the same donors. For AD, amyloid plaque–like aggregates have been reported in organoid models under extended culture conditions (30), though the conditions and timescales required are much longer than those in directly converted neurons. In HD, the failure of iPSC-derived neurons to recapitulate mutant HTT aggregation may reflect in part the requirement for somatic CAG repeat expansion to grow to lengths of 150–180 repeats that are unlikely to accumulate within the timescale of standard iPSC differentiation, regardless of the aging strategy applied. For these questions, direct conversion is not an enhancement of an existing method but a distinct experimental requirement. As conversion protocols improve in efficiency and reproducibility, direct conversion is likely to become an increasingly important complement to iPSC-based approaches, particularly for sporadic disease modeling where donor age is central to the biology.

## Conclusion

iPSC-derived models of NDDs have demonstrated that patient-specific genetic backgrounds can be studied in living human neurons. For diseases driven by high-penetrance familial mutations, these models have reproduced disease-relevant cellular phenotypes, identified convergent pathways across genetically distinct conditions, and provided mechanistic insights that are not accessible from animal models or postmortem tissue (24, 31, 136). The progression from 2D monocultures to coculture systems and 3D organoids has broadened the range of questions human cell-based models can address, enabling investigation of neuron-glia interactions, tissue-level organization, and disease phenotypes that require higher maturation levels that simpler systems cannot replicate (94, 137).

The central limitation that remains insufficiently resolved is the aging problem. Reprogramming to pluripotency erases the epigenetic age of donor cells, producing neurons with a fetal rather than adult cellular identity. Because sporadic neurodegeneration, which accounts for 85% to 95% of cases across all major conditions, is age dependent in its onset and progression, models that lack the aging context of the donor are limited in their ability to capture the biology most relevant to the patients these diseases affect. Drugs found effective in iPSC-derived neurons might not work in aged cells and tissues, especially if the drugs target pathways that change with aging. Strategies to approximate aspects of aging in iPSC-derived neurons are useful sensitization tools but do not restore the full endogenous aging program (101, 108, 109). Direct conversion preserves native aging and enables access to age-dependent phenotypes that are otherwise inaccessible (97, 130, 132) but at the cost of technical difficulty and reduced scalability compared with iPSC systems.

The appropriate choice of model depends on the biological question. Standard iPSC-derived neurons are well suited for studying the mechanisms of high-penetrance mutations, early pathogenic events, and genetic risk architecture. Aging-sensitized models are valuable when genetic vulnerabilities are latent in young neurons and require reduced cellular resilience to become expressed. Directly converted neurons are necessary when the question concerns age-dependent phenotypes or sporadic disease biology where the donor’s epigenetic age is central to the disease mechanism. No single system addresses all questions, and selecting the model to fit the question, rather than applying the most familiar or technically accessible system, is itself a scientific decision with consequences for what can be concluded.

The most important frontier that remains is sporadic disease modeling. The majority of patients with AD, PD, ALS, and related conditions do not carry a familial mutation; their disease emerges from the cumulative effects of polygenic risk, stochastic cellular events, and the biological changes of aging. Polygenic risk scores are beginning to identify individuals at elevated lifetime risk, but the cellular mechanisms by which combinations of common risk variants produce neurodegeneration in aged neurons remain largely unknown. Closing this gap, from population-level genetic association to cell-level mechanistic understanding, is the central challenge for the next generation of iPSC-based disease modeling. The tools reviewed here, particularly those that preserve or restore the aging context of the patient, are likely to help close this gap.

## Conflict of interest

SF has received research support from Genentech, Sanofi, Haleon, Altos Labs, Corsalex, Vesper Bio, Topos Bio, and Babylon Bioscience for specific research projects.

## Funding support

This work is the result of NIH funding, in whole or in part, and is subject to the NIH Public Access Policy. Through acceptance of this federal funding, the NIH has been given a right to make the work publicly available in PubMed Central.

- NIH National Institutes on Aging (R01 AG064579, P01 AG054407).
- NIH National Library of Medicine (R01 LM013617).
- NIH National Institute of Environmental Health Sciences (R01 ES036325).
- The JSRM Foundation.
- The Barry and Marie Lipman Parkinson’s Disease Breakthrough Initiative.
- California Institute for Regenerative Medicine (DISC0-16039 and DISC0-13914).
- The Veteran’s Administration (RD-24-009).
- Target ALS Foundation.
- Packard Center Project Genesis.
- Taube Philanthropies.
- Collaborative Center for XDP.
- US Department of Defense (HT9425-25-1-0191).
- NeuroVC.
- Altos Labs.
- Haleon.
- Topos Bio.
- NIH National Institute of Neurological Disorders and Stroke (R01 NS124848).
- The Koret Foundation.

## Supplementary Material

Supplemental data

## Figures and Tables

**Figure 1:**
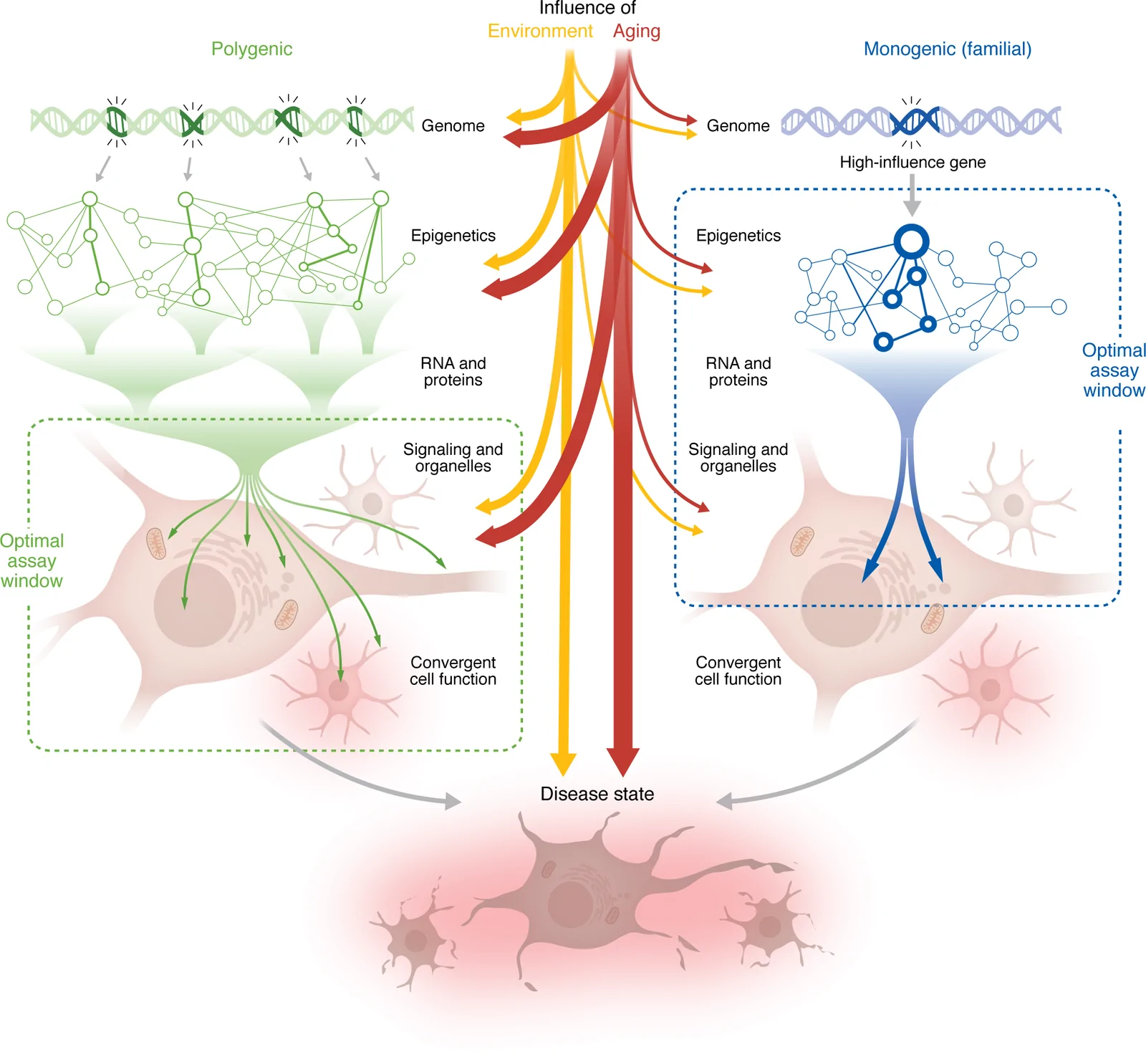
Genetic architecture determines the optimal assay window in iPSC models of neurodegeneration. The figure contrasts two disease architectures and their implications for assay design in iPSC-based studies. In polygenic (sporadic) neurodegeneration (left), many common variants each exert a small individual effect. Their signals are weak at upstream steps but accumulate at convergent downstream cellular endpoints; thus, assays placed at the convergent level provide the greatest sensitivity for detecting polygenic risk. In monogenic (familial) disease (right), a single high-penetrance variant drives a large primary effect at the molecular level. Homeostatic mechanisms progressively buffer this effect at each downstream step, so the optimal assay window lies closest to the primary molecular event. Environmental exposures (center, yellow) and cellular aging (center, red) modulate both architectures across multiple levels, including epigenetic marks, RNA and protein levels, and signaling pathways, progressively reducing cellular resilience, lowering the threshold at which accumulated damage produces a disease state.

**Figure 2:**
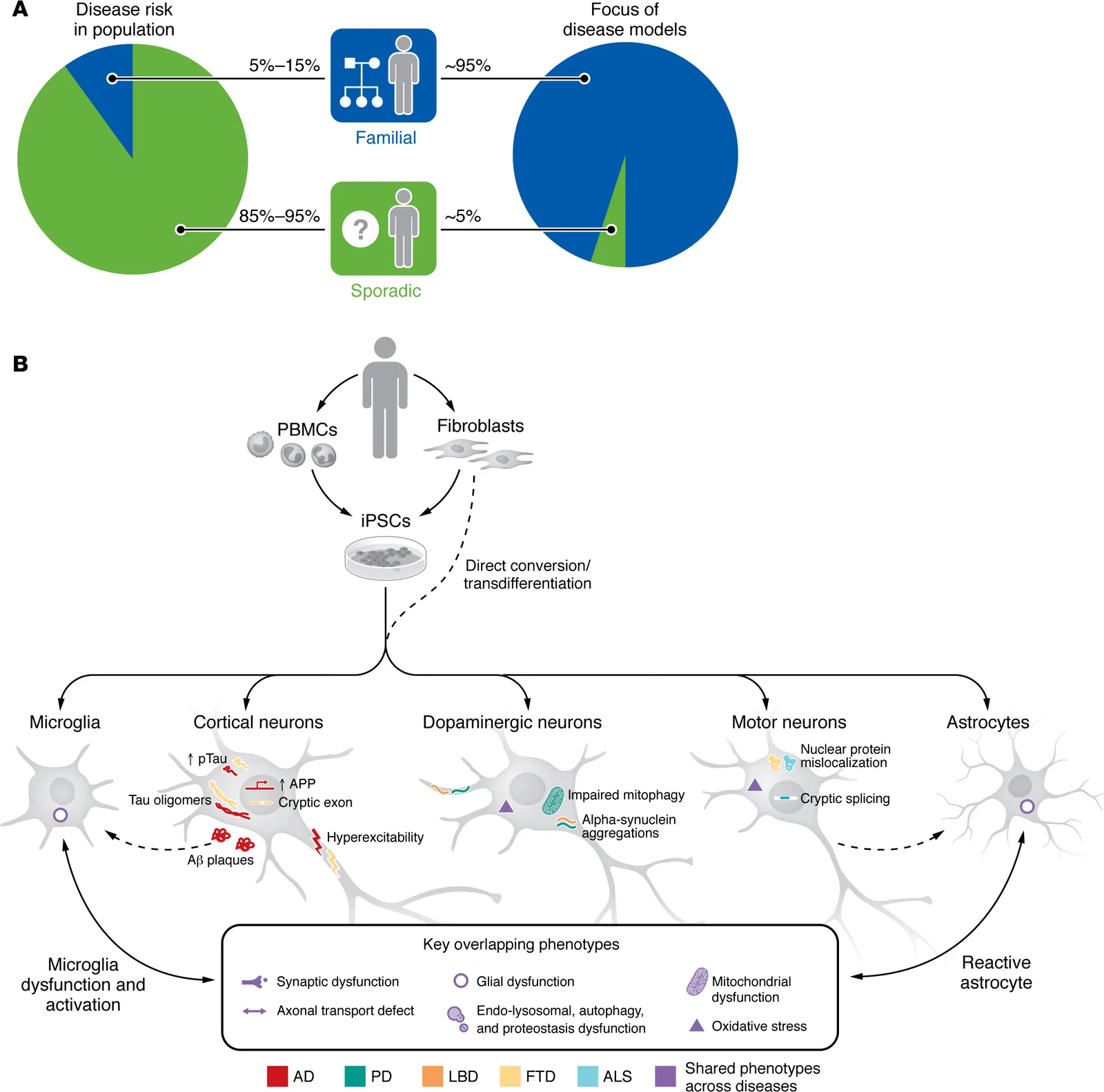
Disease-specific and convergent cellular phenotypes in iPSC-derived models of neurodegeneration. (**A**) 85%–95% of NDD cases are sporadic and 5%–15% carry a familial mutation. In contrast, approximately 95% of iPSC-based disease models to date have been built on high-penetrance familial mutations, representing a systematic gap in the field. (**B**) Patient-derived somatic cells (fibroblasts or PBMCs) can be reprogrammed to iPSCs and differentiated into disease-relevant cell types or converted directly into neurons by transdifferentiation, which preserves the donor’s epigenetic age. Five major iPSC-derived cell types are shown with their disease-associated phenotypes, including microglia, cortical neurons that include both excitatory neurons and inhibitory interneurons, dopaminergic neurons, motor neurons, and astrocytes. Color coding indicates the primary disease(s) in which each phenotype has been demonstrated with each cell type: AD (red), PD (green), LBD (orange), FTD (yellow), and ALS (blue). Lower: Key cellular phenotypes shared across multiple diseases are depicted, including synaptic dysfunction, axonal transport defects, glial dysfunction, endo-lysosomal and proteostasis dysfunction, mitochondrial dysfunction, and oxidative stress, underscoring the convergent biology of neurodegeneration across genetically distinct conditions. Aβ, amyloid-β; APP, amyloid precursor protein; PBMCs, peripheral blood mononuclear cells; pTau, phosphorylated tau.

## Version 1 10/15/2026

Electronic publication

## References

[1] Finkbeiner S (2010). Bridging the Valley of Death of therapeutics for neurodegeneration. Nat Med.

[2] Mortberg MA (2022). Disease stages and therapeutic hypotheses in two decades of neurodegenerative disease clinical trials. Sci Rep.

[3] Finkbeiner S (2020). Functional genomics, genetic risk profiling and cell phenotypes in neurodegenerative disease. Neurobiol Dis.

[4] DeJesus-Hernandez M (2011). Expanded GGGGCC hexanucleotide repeat in noncoding region of C9ORF72 causes chromosome 9p-linked FTD and ALS. Neuron.

[5] Renton AE (2011). A hexanucleotide repeat expansion in C9ORF72 is the cause of chromosome 9p21-linked ALS-FTD. Neuron.

[6] Takahashi K (2007). Induction of pluripotent stem cells from adult human fibroblasts by defined factors. Cell.

[7] Yu J (2007). Induced pluripotent stem cell lines derived from human somatic cells. Science.

[8] Gore A (2011). Somatic coding mutations in human induced pluripotent stem cells. Nature.

[9] Klim JR (2019). ALS-implicated protein TDP-43 sustains levels of STMN2, a mediator of motor neuron growth and repair. Nat Neurosci.

[10] Brown AL (2022). TDP-43 loss and ALS-risk SNPs drive mis-splicing and depletion of UNC13A. Nature.

[11] Nitsche A (2021). Alzheimer-related genes show accelerated evolution. Mol Psychiatry.

[12] Bertram L (2005). The genetic epidemiology of neurodegenerative disease. J Clin Invest.

[13] Gatz M (2006). Role of genes and environments for explaining Alzheimer disease. Arch Gen Psychiatry.

[14] Al-Chalabi A (2010). An estimate of amyotrophic lateral sclerosis heritability using twin data. J Neurol Neurosurg Psychiatry.

[15] Goldman SM (2019). Concordance for Parkinson’s disease in twins: A 20-year update. Ann Neurol.

[16] Hop PJ (2026). Large-scale exome analyses reveal new rare variant contributions in amyotrophic lateral sclerosis. Nat Genet.

[17] Lambert JC (2013). Meta-analysis of 74,046 individuals identifies 11 new susceptibility loci for Alzheimer’s disease. Nat Genet.

[18] Nalls MA (2019). Identification of novel risk loci, causal insights, and heritable risk for Parkinson’s disease: a meta-analysis of genome-wide association studies. Lancet Neurol.

[19] Ho R (2016). ALS disrupts spinal motor neuron maturation and aging pathways within gene co-expression networks. Nat Neurosci.

[20] Pantazis CB (2022). A reference human induced pluripotent stem cell line for large-scale collaborative studies. Cell Stem Cell.

[21] Ideker T (2012). Differential network biology. Mol Syst Biol.

[22] Arrasate M (2004). Inclusion body formation reduces levels of mutant huntingtin and the risk of neuronal death. Nature.

[23] Lish AM (2025). CLU alleviates Alzheimer’s disease-relevant processes by modulating astrocyte reactivity and microglia-dependent synaptic density. Neuron.

[24] Israel MA (2012). Probing sporadic and familial Alzheimer’s disease using induced pluripotent stem cells. Nature.

[25] Lagomarsino VN (2021). Stem cell-derived neurons reflect features of protein networks, neuropathology, and cognitive outcome of their aged human donors. Neuron.

[26] Hou Y (2019). Ageing as a risk factor for neurodegenerative disease. Nat Rev Neurol.

[27] Panagiotakopoulou V (2020). Interferon-γ signaling synergizes with LRRK2 in neurons and microglia derived from human induced pluripotent stem cells. Nat Commun.

[28] Weiss F (2024). Astrocytes contribute to toll-like receptor 2-mediated neurodegeneration and alpha-synuclein pathology in a human midbrain Parkinson’s model. Transl Neurodegener.

[29] Chen X (2021). Modeling sporadic Alzheimer’s disease in human brain organoids under serum exposure. Adv Sci (Weinh).

[30] Gonzalez C (2018). Modeling amyloid beta and tau pathology in human cerebral organoids. Mol Psychiatry.

[31] Nguyen HN (2011). LRRK2 mutant iPSC-derived DA neurons demonstrate increased susceptibility to oxidative stress. Cell Stem Cell.

[32] Schöndorf DC (2014). iPSC-derived neurons from GBA1-associated Parkinson’s disease patients show autophagic defects and impaired calcium homeostasis. Nat Commun.

[33] Sánchez-Danés A (2012). Disease-specific phenotypes in dopamine neurons from human iPS-based models of genetic and sporadic Parkinson’s disease. EMBO Mol Med.

[34] Almeida S (2013). Modeling key pathological features of frontotemporal dementia with C9ORF72 repeat expansion in iPSC-derived human neurons. Acta Neuropathol.

[35] Chen H (2014). Modeling ALS with iPSCs reveals that mutant SOD1 misregulates neurofilament balance in motor neurons. Cell Stem Cell.

[36] Yu CH (2020). TDP-43 triggers mitochondrial DNA release via mPTP to activate cGAS/STING in ALS. Cell.

[37] Fazal R (2021). HDAC6 inhibition restores TDP-43 pathology and axonal transport defects in human motor neurons with TARDBP mutations. EMBO J.

[38] Schork NJ (2023). Pathway-specific polygenic risk scores correlate with clinical status and Alzheimer’s disease-related biomarkers. J Alzheimers Dis.

[39] Huber N (2026). Frontotemporal dementia patient-derived iPSC neurons show cell pathological hallmarks and evidence for synaptic dysfunction and DNA damage. Mol Psychiatry.

[40] Baxi EG (2022). Answer ALS, a large-scale resource for sporadic and familial ALS combining clinical and multi-omics data from induced pluripotent cell lines. Nat Neurosci.

[41] Workman MJ (2023). Large-scale differentiation of iPSC-derived motor neurons from ALS and control subjects. Neuron.

[42] Fujimori K (2018). Modeling sporadic ALS in iPSC-derived motor neurons identifies a potential therapeutic agent. Nat Med.

[43] Coyne AN (2021). Nuclear accumulation of CHMP7 initiates nuclear pore complex injury and subsequent TDP-43 dysfunction in sporadic and familial ALS. Sci Transl Med.

[44] Namboori SC (2021). Single-cell transcriptomics identifies master regulators of neurodegeneration in SOD1 ALS iPSC-derived motor neurons. Stem Cell Reports.

[45] Bye CR (2026). Large-scale drug screening in iPSC-derived motor neurons from sporadic ALS patients identifies a potential combinatorial therapy. Nat Neurosci.

[46] Corder EH (1993). Gene dose of apolipoprotein E type 4 allele and the risk of Alzheimer’s disease in late onset families. Science.

[47] Wang C (2018). Gain of toxic apolipoprotein E4 effects in human iPSC-derived neurons is ameliorated by a small-molecule structure corrector. Nat Med.

[48] Lin YT (2018). *APOE*4 causes widespread molecular and cellular alterations associated with Alzheimer’s disease phenotypes in human iPSC-derived brain cell types. Neuron.

[49] Tcw J (2022). Cholesterol and matrisome pathways dysregulated in astrocytes and microglia. Cell.

[50] Hulihan MM (2008). LRRK2 Gly2019Ser penetrance in Arab-Berber patients from Tunisia: a case-control genetic study. Lancet Neurol.

[51] Tezuka T (2022). Pathophysiological evaluation of the LRRK2 G2385R risk variant for Parkinson’s disease. NPJ Parkinsons Dis.

[52] Gijselinck I (2018). The genetics of C9orf72 expansions. Cold Spring Harb Perspect Med.

[53] Zhang Y (2013). Rapid single-step induction of functional neurons from human pluripotent stem cells. Neuron.

[54] Nehme R (2018). Combining NGN2 programming with developmental patterning generates human excitatory neurons with NMDAR-mediated synaptic transmission. Cell Rep.

[55] Chambers SM (2009). Highly efficient neural conversion of human ES and iPS cells by dual inhibition of SMAD signaling. Nat Biotechnol.

[56] Shi Y (2012). Human cerebral cortex development from pluripotent stem cells to functional excitatory synapses. Nat Neurosci.

[57] Nicholas CR (2013). Functional maturation of hPSC-derived forebrain interneurons requires an extended timeline and mimics human neural development. Cell Stem Cell.

[58] Maroof AM (2013). Directed differentiation and functional maturation of cortical interneurons from human embryonic stem cells. Cell Stem Cell.

[59] Kriks S (2011). Dopamine neurons derived from human ES cells efficiently engraft in animal models of Parkinson’s disease. Nature.

[60] Doi D (2020). Pre-clinical study of induced pluripotent stem cell-derived dopaminergic progenitor cells for Parkinson’s disease. Nat Commun.

[61] Wichterle H (2008). Differentiation of mouse embryonic stem cells to spinal motor neurons. Curr Protoc Stem Cell Biol.

[62] Peljto M (2010). Functional diversity of ESC-derived motor neuron subtypes revealed through intraspinal transplantation. Cell Stem Cell.

[63] Shi Y (2012). Directed differentiation of human pluripotent stem cells to cerebral cortex neurons and neural networks. Nat Protoc.

[64] Krencik R (2011). Directed differentiation of functional astroglial subtypes from human pluripotent stem cells. Nat Protoc.

[65] Tcw J (2017). An efficient platform for astrocyte differentiation from human induced pluripotent stem cells. Stem Cell Reports.

[66] Douvaras P (2015). Generation and isolation of oligodendrocyte progenitor cells from human pluripotent stem cells. Nat Protoc.

[67] Abud EM (2017). iPSC-derived human microglia-like cells to study neurological diseases. Neuron.

[68] Muffat J (2016). Efficient derivation of microglia-like cells from human pluripotent stem cells. Nat Med.

[69] McQuade A (2018). Development and validation of a simplified method to generate human microglia from pluripotent stem cells. Mol Neurodegener.

[70] Guttikonda SR (2021). Fully defined human pluripotent stem cell-derived microglia and tri-culture system model C3 production in Alzheimer’s disease. Nat Neurosci.

[71] Toda Robert A (2026). Comparative lipidomics of iPSC-derived microglia protocols reveal lipid droplet and immune differences mediated by media composition. Stem Cell Reports.

[72] Dräger NM (2022). A CRISPRi/a platform in human iPSC-derived microglia uncovers regulators of disease states. Nat Neurosci.

[73] Di Giorgio FP (2008). Human embryonic stem cell-derived motor neurons are sensitive to the toxic effect of glial cells carrying an ALS-causing mutation. Cell Stem Cell.

[74] Serio A (2013). Astrocyte pathology and the absence of non-cell autonomy in an induced pluripotent stem cell model of TDP-43 proteinopathy. Proc Natl Acad Sci U S A.

[75] Meyer K (2014). Direct conversion of patient fibroblasts demonstrates non-cell autonomous toxicity of astrocytes to motor neurons in familial and sporadic ALS. Proc Natl Acad Sci U S A.

[76] Supakul S (2024). Mutual interaction of neurons and astrocytes derived from iPSCs with APP V717L mutation developed the astrocytic phenotypes of Alzheimer’s disease. Inflamm Regen.

[77] Lish AM (2025). Astrocyte induction of disease-associated microglia is suppressed by acute exposure to fAD neurons in human iPSC triple cultures. Cell Rep.

[78] Ryan SK (2020). Protocol for tri-culture of hiPSC-derived neurons, astrocytes, and microglia. STAR Protoc.

[79] Madhavan M (2018). Induction of myelinating oligodendrocytes in human cortical spheroids. Nat Methods.

[80] Paşca AM (2015). Functional cortical neurons and astrocytes from human pluripotent stem cells in 3D culture. Nat Methods.

[81] Sloan SA (2017). Human astrocyte maturation captured in 3D cerebral cortical spheroids derived from pluripotent stem cells. Neuron.

[82] Miguel L (2019). Detection of all adult Tau isoforms in a 3D culture model of iPSC-derived neurons. Stem Cell Res.

[83] Choi SH (2014). A three-dimensional human neural cell culture model of Alzheimer’s disease. Nature.

[84] de Majo M (2023). Granulin loss of function in human mature brain organoids implicates astrocytes in TDP-43 pathology. Stem Cell Reports.

[85] Bossolasco P (2022). GRN-/- iPSC-derived cortical neurons recapitulate the pathological findings of both frontotemporal lobar degeneration and neuronal ceroidolipofuscinosis. Neurobiol Dis.

[86] Gordon A (2021). Long-term maturation of human cortical organoids matches key early postnatal transitions. Nat Neurosci.

[87] He Z (2024). An integrated transcriptomic cell atlas of human neural organoids. Nature.

[88] Camp JG (2015). Human cerebral organoids recapitulate gene expression programs of fetal neocortex development. Proc Natl Acad Sci U S A.

[89] Giandomenico SL (2019). Cerebral organoids at the air-liquid interface generate diverse nerve tracts with functional output. Nat Neurosci.

[90] Cakir B (2019). Engineering of human brain organoids with a functional vascular-like system. Nat Methods.

[91] Kalpana K (2025). Generating Neuroimmune Assembloids Using Human Induced Pluripotent Stem Cell (iPSC)-Derived Cortical Organoids and Microglia. Methods Mol Biol.

[92] Andersen J (2020). Generation of functional Human 3D cortico-motor assembloids. Cell.

[93] Mansour AA (2018). An in vivo model of functional and vascularized human brain organoids. Nat Biotechnol.

[94] Revah O (2022). Maturation and circuit integration of transplanted human cortical organoids. Nature.

[95] Kawas C (2000). Age-specific incidence rates of Alzheimer’s disease: the Baltimore Longitudinal Study of Aging. Neurology.

[96] López-Otín C (2013). The hallmarks of aging. Cell.

[97] Mertens J (2015). Directly reprogrammed human neurons retain aging-associated transcriptomic signatures and reveal age-related nucleocytoplasmic defects. Cell Stem Cell.

[98] Scaffidi P (2006). Lamin A-dependent nuclear defects in human aging. Science.

[99] Kudlow BA (2006). Aging: progeria and the lamin connection. Curr Biol.

[100] Liu GH (2011). Recapitulation of premature ageing with iPSCs from Hutchinson-Gilford progeria syndrome. Nature.

[101] Miller JD (2013). Human iPSC-based modeling of late-onset disease via progerin-induced aging. Cell Stem Cell.

[102] Sinha JK (2014). Progeria: a rare genetic premature ageing disorder. Indian J Med Res.

[103] Oh HS (2023). Organ aging signatures in the plasma proteome track health and disease. Nature.

[104] Schaum N (2020). Ageing hallmarks exhibit organ-specific temporal signatures. Nature.

[105] von Zglinicki T (2002). Oxidative stress shortens telomeres. Trends Biochem Sci.

[106] Rudolph KL (1999). Longevity, stress response, and cancer in aging telomerase-deficient mice. Cell.

[107] Jaskelioff M (2011). Telomerase reactivation reverses tissue degeneration in aged telomerase-deficient mice. Nature.

[108] Vera E (2016). Generating late-onset human iPSC-based disease models by inducing neuronal age-related phenotypes through telomerase manipulation. Cell Rep.

[109] Saurat N (2024). Genome-wide CRISPR screen identifies neddylation as a regulator of neuronal aging and AD neurodegeneration. Cell Stem Cell.

[110] Zhang S (2024). Protein neddylation and its role in health and diseases. Signal Transduct Target Ther.

[111] Fathi A (2022). Chemically induced senescence in human stem cell-derived neurons promotes phenotypic presentation of neurodegeneration. Aging Cell.

[112] McHugh D (2018). Senescence and aging: Causes, consequences, and therapeutic avenues. J Cell Biol.

[113] Ogrodnik M (2021). Cellular aging beyond cellular senescence: Markers of senescence prior to cell cycle arrest in vitro and in vivo. Aging Cell.

[114] Schmeer C (2019). Dissecting aging and senescence-current concepts and open lessons. Cells.

[115] Suryadevara V (2026). Charting human cellular senescence in aging and disease. Cell.

[116] López-Otín C (2023). Hallmarks of aging: An expanding universe. Cell.

[117] Herdy JR (2024). Neuronal senescence may drive brain aging. Science.

[118] Ishikawa S (2020). Proteostasis failure and cellular senescence in long-term cultured postmitotic rat neurons. Aging Cell.

[119] Chen J (2025). Transcriptional regulation by PHGDH drives amyloid pathology in Alzheimer’s disease. Cell.

[120] Kluever V (2022). Protein lifetimes in aged brains reveal a proteostatic adaptation linking physiological aging to neurodegeneration. Sci Adv.

[121] Huh CJ (2016). Maintenance of age in human neurons generated by microRNA-based neuronal conversion of fibroblasts. Elife.

[122] Yoo AS (2011). MicroRNA-mediated conversion of human fibroblasts to neurons. Nature.

[123] Victor MB (2014). Generation of human striatal neurons by microRNA-dependent direct conversion of fibroblasts. Neuron.

[124] Son EY (2011). Conversion of mouse and human fibroblasts into functional spinal motor neurons. Cell Stem Cell.

[125] Vierbuchen T (2010). Direct conversion of fibroblasts to functional neurons by defined factors. Nature.

[126] Pang ZP (2011). Induction of human neuronal cells by defined transcription factors. Nature.

[127] Caiazzo M (2011). Direct generation of functional dopaminergic neurons from mouse and human fibroblasts. Nature.

[128] Victor MB (2018). Striatal neurons directly converted from Huntington’s disease patient fibroblasts recapitulate age-associated disease phenotypes. Nat Neurosci.

[129] Oh YM (2022). Age-related Huntington’s disease progression modeled in directly reprogrammed patient-derived striatal neurons highlights impaired autophagy. Nat Neurosci.

[130] Capano LS (2022). Recapitulation of endogenous 4R tau expression and formation of insoluble tau in directly reprogrammed human neurons. Cell Stem Cell.

[131] Parra Bravo C (2024). Human iPSC 4R tauopathy model uncovers modifiers of tau propagation. Cell.

[132] Chou CC (2025). Proteostasis and lysosomal repair deficits in transdifferentiated neurons of Alzheimer’s disease. Nat Cell Biol.

[133] Mertens J (2021). Age-dependent instability of mature neuronal fate in induced neurons from Alzheimer’s patients. Cell Stem Cell.

[134] Arbab M (2014). Modeling motor neuron disease: the matter of time. Trends Neurosci.

[135] Smith MC (2013). hTERT extends the life of human fibroblasts without compromising type I interferon signaling. PLoS One.

[136] Kondo T (2013). Modeling Alzheimer’s disease with iPSCs reveals stress phenotypes associated with intracellular Aβ and differential drug responsiveness. Cell Stem Cell.

[137] Park J (2018). A 3D human triculture system modeling neurodegeneration and neuroinflammation in Alzheimer’s disease. Nat Neurosci.

