# Supplementary material for: Modeling human neurodegeneration with iPSCs: opportunities, limitations, and the challenge of aging
Source: J Clin Invest. 2026 Oct 15;136(20):e209602. doi: 10.1172/JCI209602 (PMC13668611; doi:10.1172/JCI209602)
Supplement: Supplementary file 1 [file jci-136-209602-s288.pdf]

Online supplementary material regarding the review manuscript

Modeling human neurodegeneration with iPSCs: opportunities, limitations, and the challenge of aging

Shijie Wang[1,2], Lisa Elia[1,2], Weiyi Liu[1,2,3], Steven Finkbeiner[1,2,4]

Affiliations:

1. Center for Systems and Therapeutics, Gladstone Institutes, San Francisco, CA 94158, USA
2. Gladstone Institutes, San Francisco, CA 94158, USA
3. Department of Metabolic Biology and Nutrition, University of California, Berkeley, Berkeley, CA, USA; Molecular Toxicology Graduate Program, University of California, Berkeley, Berkeley, CA, USA
4. Department of Neurology, University of California, San Francisco, CA 94158, USA; Department of Physiology, University of California, San Francisco, CA 94158, USA; Neuroscience Graduate Program and Biomedical Sciences Program, University of California, San Francisco, CA 94158, USA

Table 1. Alzheimer disease (AD) phenotypes recapitulated in iPSC-derived models

Genetic variants modeled	Cell types	Key phenotypes recapitulated
Familial: APP (duplications, Swedish, London) (1-13)	Forebrain excitatory neurons (1, 3-7, 9, 11); Co-culture/Tri-culture (12); 3D organoids/spheroids (2, 8, 10)	Increased APP mRNA, processing defects & β-CTF accumulation (1, 3, 6); Increased Aβ40, Aβ42 secretion or Aβ42/Aβ40 ratio (1, 2, 6, 8, 10); Enhanced amyloid-β accumulation, extracellular deposits & plaque-like pathology (2); Increased GSK-3β & BACE levels (1); Tau hyperphosphorylation or aggregation (2, 9-11); Impaired endo-lysosomal trafficking (2, 3, 6); Autophagy dysfunction & synapse loss (10); Mitochondrial dysfunction & metabolic stress (3, 13); Synaptic dysfunction & neuronal vulnerability (10, 12); Altered microglial response (12); Astrocyte degeneration & inflammation response (12)
Familial: PSEN1 (M146L, A246E, multiple) (1-3, 5, 6, 10, 13-16)	Forebrain excitatory neurons (1, 3, 5, 6, 14, 15); Cortical organoids (14); 3D organoids/spheroids (2, 10); Astrocytes (16)	Increased APP mRNA, processing defects & β-CTF accumulation (1, 3, 6); Increased Aβ40, Aβ42 secretion or Aβ42/Aβ40 ratio (1, 2, 6, 10, 15); Enhanced amyloid-β accumulation, extracellular deposits & plaque-like pathology (2); Increased GSK-3β & BACE levels (1); Tau hyperphosphorylation or aggregation (2, 10); Impaired endo-lysosomal trafficking (2, 3, 6); Autophagy dysfunction & synapse loss (10); Mitochondrial dysfunction & metabolic stress (3, 13); Synaptic dysfunction & neuronal vulnerability (10); Premature neurogenesis (14); Astrocyte degeneration & inflammation response (16)
Familial: PSEN2 (N141I, multiple) (1, 5, 17, 18)	Forebrain excitatory neurons (1, 5, 18); Cholinergic neurons (17)	Increased APP mRNA, processing defects & β-CTF accumulation (1); Increased Aβ40, Aβ42 secretion or Aβ42/Aβ40 ratio (1, 17); Increased GSK-3β & BACE levels (1); Mitochondrial dysfunction & metabolic stress (18)
Familial: Down Syndrome (19-25)	Forebrain excitatory neurons (19, 22, 24); 3D organoids/spheroids (20, 21, 25); iPSC neuron-mouse hippocampus chimeric model (23)	Increased Aβ40, Aβ42 secretion or Aβ42/Aβ40 ratio (20, 22, 24); Enhanced amyloid-β accumulation, extracellular deposits & plaque-like pathology (20, 21); Increased GSK-3β & BACE levels (21); Tau hyperphosphorylation or aggregation (20-22, 24); Retromer complex dysfunction (24); Synaptic dysfunction & neuronal vulnerability (19, 20); Synaptic/neuronal gene network dysregulation (22); Altered microglial response (23); Neuroinflammation (23)
Risk factor: APOE ε4 (26-35)	GABAergic neurons (26); Cortical organoids (28, 29); Co-culture/Tri-culture (33); Astrocytes (30, 31, 34); Microglia (27); Oligodendroglia (35); iPSC neuron-mouse hippocampus chimeric model (32)	Increased APP mRNA, processing defects & β-CTF accumulation (26); Increased Aβ40, Aβ42 secretion or Aβ42/Aβ40 ratio (27); Enhanced amyloid-β accumulation, extracellular deposits & plaque-like pathology (32); Tau hyperphosphorylation or aggregation (26, 27, 32); Impaired endo-lysosomal trafficking (27); Autophagy dysfunction & synapse loss (31); Mitochondrial dysfunction & metabolic stress (31); APOE4-dependent cholesterol metabolism defects (26, 27, 31); APOE4 genomic & chromatin dysregulation in oligodendroglia (35); Synaptic dysfunction & neuronal vulnerability (26); Synaptic/neuronal gene network dysregulation (28); Altered microglial response (27, 32-34); Astrocyte degeneration & inflammation response (27, 31, 33, 34)
Risk factor: TREM2 (R47H) (33, 36, 37)	Co-culture/Tri-culture (33); Microglia (36, 37)	APOE4-dependent cholesterol metabolism defects (36); Altered microglial response (33, 36, 37); Astrocyte degeneration & inflammation response (33)
Risk factor: SORL1 (haploinsufficiency) (38-40)	Forebrain excitatory neurons (38, 39); Microglia (40)	Increased APP mRNA, processing defects & β-CTF accumulation (38, 39); Increased Aβ40, Aβ42 secretion or Aβ42/Aβ40 ratio (39); Impaired endo-lysosomal trafficking (38-40); APOE4-dependent cholesterol metabolism defects (39)
Risk factor: PLCG2 (protective P522R and AD-risk variants) (41)	Microglia (41)	Altered microglial response (41)
Risk factor: ABCA7 LoF (42)	Forebrain excitatory neurons (42); Cortical organoids (42)	Mitochondrial dysfunction & metabolic stress (42); APOE4-dependent cholesterol metabolism defects (42)
Risk factor: PITRM1 LoF (43)	3D organoids/spheroids (43)	Increased Aβ40, Aβ42 secretion or Aβ42/Aβ40 ratio (43); Tau hyperphosphorylation or aggregation (43); Mitochondrial dysfunction & metabolic stress (43)
Sporadic (1, 28, 44-46)	Forebrain excitatory neurons (1, 44); Cortical organoids (28, 45); 3D organoids/spheroids (46)	Increased APP mRNA, processing defects & β-CTF accumulation (1); Increased Aβ40, Aβ42 secretion or Aβ42/Aβ40 ratio (1, 44-46); Enhanced amyloid-β accumulation, extracellular deposits & plaque-like pathology (46); Increased GSK-3β & BACE levels (1, 45); Tau hyperphosphorylation or aggregation (44-46); Synaptic dysfunction & neuronal vulnerability (45, 46); Synaptic/neuronal gene network dysregulation (28); Altered microglial response (46); Astrocyte degeneration & inflammation response (45); Neuroinflammation (46)

Abbreviations: LoF, loss of function; β-CTF, beta-C-terminal fragment; Aβ, amyloid-beta; BACE, beta-site APP-cleaving enzyme; GSK-3β, glycogen synthase kinase 3β.

Table 1 lists disease-relevant phenotypes recapitulated in iPSC-derived models of Alzheimer disease. For each study, the table provides genetic variants and risk factors modeled, principal cell types generated, and key phenotypes demonstrated, with supporting references. 'Sporadic' designates studies that included iPSC lines derived from patients without a known familial mutation. Phenotypes were observed under standard 2D monoculture conditions, with or without exogenous stress, or in 3D organoid systems.

Table 2. Parkinson disease (PD) and Lewy body dementia (LBD) phenotypes recapitulated in iPSC-derived models

Genetic variants modeled	Cell types	Key phenotypes recapitulated
Familial: SNCA (A53T, A30P, triplication) (47-59)	Midbrain dopaminergic neurons (47-52, 54, 58); Cortical neurons (56, 57); Midbrain organoids (53, 55, 59)	α-synuclein accumulation, propagation & Lewy body-like inclusion formation (47, 48, 51, 54); Tau deposition (LRRK2) (57); Mitochondrial dysfunction & impaired mitophagy (51, 57); Endo-lysosomal, autophagy & proteostasis dysfunction (54, 57); Enhanced oxidative stress or sensitivity to oxidative stress (48, 51); Dysregulation of Ca^{2+} homeostasis (51); Synaptic activity or vesicle cycling impairment (56)
Familial: LRRK2 (G2019S, R1441C) (57, 60-71)	Midbrain dopaminergic neurons (60-65, 69, 70, 72); Cortical neurons (57); Astrocytes (68); Microglia (67, 71)	α-synuclein accumulation, propagation & Lewy body-like inclusion formation (60, 61); Tau deposition (LRRK2) (57); Mitochondrial dysfunction & impaired mitophagy (57, 64); Endo-lysosomal, autophagy & proteostasis dysfunction (57, 61); Enhanced oxidative stress or sensitivity to oxidative stress (60); Epigenic dysregulation (LRRK2, sporadic PD) (62); Elevated LRRK2 kinase activity (70, 71); Dopaminergic neuron vulnerability (60, 61, 69); Astrocytic atrophy (LRRK2) & reduced astrocytic reactivity (PRKN) (68); IFN-γ/LRRK2 synergistic neuroinflammatory vulnerability in DA neurons & microglia (67)
Familial: PARKN (deletions/mutations) (57, 63, 73-77)	Midbrain dopaminergic neurons (63, 73, 75); Cortical neurons (57); Midbrain organoids (74, 76); Astrocytes (74, 76, 77)	α-synuclein accumulation, propagation & Lewy body-like inclusion formation (73, 76); Tau deposition (LRRK2) (57); Mitochondrial dysfunction & impaired mitophagy (57, 73, 75); Endo-lysosomal, autophagy & proteostasis dysfunction (57, 76); Astrocyte-driven dopaminergic neurodegeneration (77); Astrocytic atrophy (LRRK2) & reduced astrocytic reactivity (PRKN) (74, 76)
Familial: PINK1 (mutations) (73, 78)	Midbrain dopaminergic neurons (73, 78)	α-synuclein accumulation, propagation & Lewy body-like inclusion formation (73); Mitochondrial dysfunction & impaired mitophagy (73); Synaptic activity or vesicle cycling impairment (78)
Familial: GBA (N370S, L444P) (79-83)	Midbrain dopaminergic neurons (79-81, 83); Midbrain organoids (82)	α-synuclein accumulation, propagation & Lewy body-like inclusion formation (79, 81, 83); Endo-lysosomal, autophagy & proteostasis dysfunction (79, 81); Enhanced oxidative stress or sensitivity to oxidative stress (82); Dysregulation of Ca^{2+} homeostasis (79); Dopaminergic neuron vulnerability (79, 82)
Familial: DJ-1 (mutations) (84)	Midbrain dopaminergic neurons (84)	Enhanced oxidative stress or sensitivity to oxidative stress (84); Dopaminergic neuron vulnerability (84)
Familial: VPS35 (D620N) (85)	Midbrain dopaminergic neurons (85)	Elevated LRRK2 kinase activity (85)
Risk factor: RHOT1 (heterozygous variants) (86)	Midbrain organoids (86)	α-synuclein accumulation, propagation & Lewy body-like inclusion formation (86); Mitochondrial dysfunction & impaired mitophagy (86); Dopaminergic neuron vulnerability (86)
Sporadic (61, 62, 84, 87)	Midbrain dopaminergic neurons (61, 62, 84)	α-synuclein accumulation, propagation & Lewy body-like inclusion formation (61); Endo-lysosomal, autophagy & proteostasis dysfunction (61, 87); Enhanced oxidative stress or sensitivity to oxidative stress (84); Epigenic dysregulation (LRRK2, sporadic PD) (62); Dopaminergic neuron vulnerability (61, 84)

Abbreviations: DA, dopaminergic.

Table 2 lists disease-relevant phenotypes recapitulated in iPSC-derived models of Parkinson disease and Lewy body dementia. For each study, the table provides genetic variants and risk factors modeled, principal cell types generated, and key phenotypes demonstrated, with supporting references. ‘Sporadic’ designates studies that included iPSC lines derived from patients without a known familial mutation. Phenotypes were observed under standard 2D monoculture conditions, with or without exogenous stress, or in 3D organoid systems.

Table 3. Amyotrophic lateral sclerosis (ALS) phenotypes recapitulated in iPSC-derived models

Genetic variants modeled	Cell types	Key phenotypes recapitulated
Familial: C9orf72 (GGGGCC expansion) (88-100)	Spinal motor neurons (90, 91, 98, 99); Cortical motor neurons (88, 89, 91-93, 95); Microglia (96, 97, 100); Cerebral organoid slices (94); Neural Organoids-microglia co-culture (100)	DPR protein accumulation and cell-to-cell transmission (88, 92); RNA foci (sense & antisense) (88, 92); Cryptic splicing, intron retention & STMN2 loss (90); Nuclear protein mis-localization (TDP-43, Ran, NPC) (90); Impaired DNA damage repair (95); Axonal transport deficits or degeneration (95); Endo-lysosomal, autophagy & proteostasis dysfunction (96, 99); Motor neuron selective vulnerability & synaptic abnormalities (89, 93, 98); Astrocyte toxicity (94, 96); Microglial dysfunction, pro-inflammatory activation and impaired phagocytosis (96, 97, 100)
Familial: SOD1 (A4V, D90A, multiple) (72, 89, 91, 101-107)	Spinal motor neurons (72, 91, 101, 102, 105-107); Cortical motor neurons (89, 91); Sensorimotor organoids (with NMJ) (104); Astrocytes (103)	Neurofilament aggregation and NF-L mRNA instability (101); Cryptic splicing, intron retention & STMN2 loss (102); Axonal transport deficits or degeneration (101); Endo-lysosomal, autophagy & proteostasis dysfunction (106); Mitochondrial dysfunction & oxidative stress (107); Motor neuron selective vulnerability & synaptic abnormalities (89, 101); NMJ disruption and dysfunction (104); Disrupted motor neuron maturation (107)
Familial: TARDBP (M337V, A382T, multiple) (98, 108-117)	Spinal motor neurons (98, 108, 109, 111-115, 117); Astrocytes (110, 116)	TDP-43 aggregation and mislocalisation (110-116); Cryptic splicing, intron retention & STMN2 loss (109, 114); Axonal transport deficits or degeneration (112); Mitochondrial dysfunction & oxidative stress (108); Stress granule dysregulation & integrated stress response activation (111); Activation of cGAS/STING pathway (108); Motor neuron selective vulnerability & synaptic abnormalities (98, 110, 117); NMJ disruption and dysfunction (113); Astrocyte toxicity (110, 116)
Familial: FUS (R521C, P525L, multiple) (89, 91, 102, 118-123)	Spinal motor neurons (91, 102, 118-120, 122); Cortical motor neurons (89, 91); Motor neurons with microfluid device (123); Astrocytes (121)	Cryptic splicing, intron retention & STMN2 loss (102); Axonal transport deficits or degeneration (120, 123); Mitochondrial dysfunction & oxidative stress (123); Stress granule dysregulation & integrated stress response activation (122); Motor neuron selective vulnerability & synaptic abnormalities (89, 120); Astrocyte toxicity (121)
Familial: VCP mutations (102, 124-128)	Spinal motor neurons (102, 125, 126); Cortical motor neurons (124, 128); Microglia (127)	DPR protein accumulation and cell-to-cell transmission (124); RNA foci (sense & antisense) (124); Cryptic splicing, intron retention & STMN2 loss (102); VCP-dependent nuclear pore complex quality control defects (125); Endo-lysosomal, autophagy & proteostasis dysfunction (127, 128); Microglial dysfunction, pro-inflammatory activation and impaired phagocytosis (127)
Familial: PFN1 (profilin-1) mutations (129)	Microglia (129)	Microglial dysfunction, pro-inflammatory activation and impaired phagocytosis (129)
Risk factor: ATXN2 intermediate polyglutamine expansions (ALS risk factor) (130)	Spinal motor neurons (130)	Mitochondrial dysfunction & oxidative stress (130); Stress granule dysregulation & integrated stress response activation (130); Motor neuron selective vulnerability & synaptic abnormalities (130); Microglial dysfunction, pro-inflammatory activation and impaired phagocytosis (130)
Sporadic (90, 91, 107, 131)	Spinal motor neurons (90, 91, 107, 131); Cortical motor neurons (91)	Cryptic splicing, intron retention & STMN2 loss (90); Nuclear protein mis-localization (TDP-43, Ran, NPC) (90); Mitochondrial dysfunction & oxidative stress (107); Disrupted motor neuron maturation (107)

Abbreviations: NMJ, neuromuscular junction; DPR, dipeptide repeat protein; NF-L, neurofilament light chain; STMN2, stathmin-2; NPC, nuclear pore complex; STING, stimulator of interferon genes.

Table 3 lists disease-relevant phenotypes recapitulated in iPSC-derived models of amyotrophic lateral sclerosis. For each study, the table provides genetic variants and risk factors modeled, principal cell types generated, and key phenotypes demonstrated, with supporting references. ‘Sporadic’ designates studies that included iPSC lines derived from patients without a known familial mutation. Phenotypes were observed under standard 2D monoculture conditions, with or without exogenous stress, or in 3D organoid and organ-on-a-chip systems.

Table 4. Frontotemporal dementia (FTD) phenotypes recapitulated in iPSC-derived models

Genetic variants modeled	Cell types	Key phenotypes recapitulated
Familial: C9ORF72 repeat expansion (88, 92-96, 99, 132)	Cortical neurons (frontal/temporal) (88, 92, 93, 95, 99, 132); Cerebral organoids (MAPT V337M) (94); Microglia (96)	TDP-43 pathology (inclusions, mislocalisation) (132); DPR protein production and transmission (88, 92); RNA foci (sense & antisense) (88); Endo-lysosomal, autophagy & proteostasis dysfunction (88); DNA damage (132); Neuronal hyperexcitability, vulnerability, and synaptic dysfunction (93, 132); Microglial phagocytic & immune dysfunction (96)
Familial: MAPT (P301L, V337M, multiple) (7, 11, 133-142)	Cortical neurons (frontal/temporal) (7, 11, 134-141); Cerebral organoids (MAPT V337M) (133); Microglia (142)	Tau hyperphosphorylation, accumulation and altered 4R:3R ratio (133, 135-139, 141); Endo-lysosomal, autophagy & proteostasis dysfunction (133, 141); Mitochondrial dysfunction & oxidative stress (7, 137, 140); Nucleocytoplasmic transport defect (135); Increased stress granule formation (133); Neuronal hyperexcitability, vulnerability, and synaptic dysfunction (7, 133, 134, 137, 138, 140, 141); Microglial phagocytic & immune dysfunction (142)
Familial: TARDBP mutations (109, 114, 116)	Cortical neurons (frontal/temporal) (109, 114); Astrocytes (116)	TDP-43 pathology (inclusions, mislocalisation) (109, 114, 116); Cryptic exon inclusion (UNC13A, STMN2) (109, 114); Astrocyte toxicity (116)
Familial: GRN (haploinsufficiency) (143-146)	Cortical neurons (frontal/temporal) (144, 146); Cerebral organoids (MAPT V337M) (145); Microglia (143)	TDP-43 pathology (inclusions, mislocalisation) (144, 145); Endo-lysosomal, autophagy & proteostasis dysfunction (143, 144); Mitochondrial dysfunction & oxidative stress (143); Neuronal hyperexcitability, vulnerability, and synaptic dysfunction (145); Astrocyte toxicity (145); Microglial phagocytic & immune dysfunction (145)
Familial: VCP mutations (13, 127, 128)	Cortical neurons (frontal/temporal) (13, 128); Microglia (127)	Tau hyperphosphorylation, accumulation and altered 4R:3R ratio (128); Mitochondrial dysfunction & metabolic stress (13); Endo-lysosomal, autophagy & proteostasis dysfunction (128); Microglial phagocytic & immune dysfunction (127)
Sporadic (132)	Cortical neurons (frontal/temporal) (132)	TDP-43 pathology (inclusions, mislocalisation) (132); DNA damage (132); Neuronal hyperexcitability, vulnerability, and synaptic dysfunction (132)

Abbreviations: GRN, granulin; VCP, valosin-containing protein; DPR, dipeptide repeat protein.

Table 4 lists disease-relevant phenotypes recapitulated in iPSC-derived models of frontotemporal dementia. For each study, the table provides genetic variants and risk factors modeled, principal cell types generated, and key phenotypes demonstrated, with supporting references. ‘Sporadic’ designates studies that included iPSC lines derived from patients without a known familial mutation. Phenotypes were observed under standard 2D monoculture conditions, with or without exogenous stress, or in 3D organoid systems.

Table 5. Direct conversion strategies for modeling age-dependent neurodegeneration

Disease	Transdifferentiation approach	Genetic variants modeled	Cell types	Key phenotypes recapitulated (absent in iPSC models)
Alzheimer Disease (AD)	Small-molecule Cocktail (transgene-free) (147)	APP (147); PSEN1 (147)	Glutamatergic / pan-neuronal	Elevated Aβ42/Aβ40 ratio (higher compare to iPSC-derived neurons) (147); Tau hyperphosphorylation (147); age epigenetic signatures retained (DNA methylation clock, RanBP17 loss, nucleocytoplasmic transport defects) (147)
Alzheimer Disease (AD)	Overexpression of NGN2 + ASCL1 (148-150)	APP (148); PSEN1 (148, 149); Sporadic (149, 150)	Glutamatergic / pan-neuronal	Elevated Aβ42/Aβ40 ratio (higher compare to iPSC-derived neurons) (148-150); Tau hyperphosphorylation (148); Elevated ROS (149); DNA damage & aneuploid DNA (149, 150); Hypo-mature neuronal identity or senescence-like state (149, 150); age epigenetic signatures retained (DNA methylation clock, RanBP17 loss, nucleocytoplasmic transport defects) (148); epigenetic erosion pattern mirrors post-mortem AD brain (iPSC-neurons show no AD signature) (149, 150)
Alzheimer Disease (AD)	Overexpression of BRN2 + ASCL1 + MYT1L + NGN2 (BAMN) (151)	PSEN1 (151); Sporadic (151)	Glutamatergic / pan-neuronal	Elevated Aβ42/Aβ40 ratio (higher compare to iPSC-derived neurons) (151); Tau hyperphosphorylation (151); Endo-lysosomal, autophagy & proteostasis dysfunction (151)
Parkinson Disease (PD)	Overexpression of ASCL1 + NURR1 + LMX1A (152); Overexpression of MASH1 + NGN2 + SOX2 + NURR1 + PITX3 (153)	NA	Dopaminergic Neurons	Dopamine release; Functional action potentials; Symptom relief in PD mouse transplant model (152, 153)
Parkinson Disease (PD)	Overexpression of ASCL1 + BRN2 + MYT1L+ LMX1A + FOXA2; lentiviral (154)	NA	Dopaminergic Neurons	Dopamine release; Functional action potentials; Symptom relief in PD mouse transplant model (154)
Amyotrophic Lateral Sclerosis (ALS)	Overexpression of NGN2 + ISL1 + LHX3 (155)	FUS mutation (155)	Motor Neurons	Reduced motor neuron survival; soma shrinkage; hypo-activity; failure to form NMJs (155); SOD1/ C9orf72/ TDP-43: Increased mitochondrial membrane potential; TDP-43: mitochondrial morphology & motility defects; FUS: Reduced ATP/ADP ratio; hypermetabolic state; Neuronal Vulnerability mutation-specific survival reduction, FUS mislocalization (155)
Amyotrophic Lateral Sclerosis (ALS)	Overexpression of miR-9/9*-124 + ISL1 + LHX3 (156)	SOD1/ C9orf72/ TDP-43/ CHCHD10 mutation (156); FUS mutation (156)	Motor Neurons	Mitochondrial & metabolic dysfunction (156)
Tauopathy (Primary)	Overexpression of miR-9/9*-124 + MYT1L + ISX9; lentiviral (157)		Glutamatergic Neurons	Endogenous 4R tau expression recapitulated; insoluble tau inclusions form spontaneously; mature tau splicing; age-associated defects (157)
Huntington's Disease (HD)	Overexpression of miR-9/9*-124 + CTIP2 + DLX1/2 + MYT1L (158, 159)	HTT mutation (158, 159)	Striatal Neurons	Nuclear huntingtin protein aggregates (absent in iPSC-derived neurons from same donors) (158, 159); age-related autophagy decline; neurodegeneration recapitulated; age epigenome preserved (elevated oxidative stress, DNA damage, telomere shortening) (158)

Abbreviations: BAMN, BRN2 + ASCL1 + MYT1L + NGN2 reprogramming cocktail; Aβ, amyloid-beta; miR, microRNA; HD, Huntington disease; NMJ, neuromuscular junction; HTT, huntingtin.

Table 5 summarizes direct conversion (transdifferentiation) strategies used to model neurodegenerative diseases in patient-derived neurons, bypassing pluripotent reprogramming and thereby preserving the epigenetic age and transcriptomic signatures of donor cells. For each disease, the table provides the reprogramming approach, genetic variants or patient backgrounds studied, principal neuronal subtypes generated, and key disease-relevant phenotypes demonstrated. Phenotypes recapitulated in directly converted neurons but absent or markedly attenuated in iPSC-derived neurons from the same donors are highlighted; these phenotypes underscore the unique advantage of direct conversion for accessing age-dependent disease biology.

References Cited:

1. Israel MA, et al. Probing sporadic and familial Alzheimer's disease using induced pluripotent stem cells. *Nature*. 2012;482(7384):216–220.
2. Raja WK, et al. Self-organizing 3D human neural tissue serived from induced pluripotent stem cells recapitulate Alzheimer's disease phenotypes. *PloS one*. 2016;11(9):e0161969.
3. Woodruff G, et al. Defective transcytosis of APP and lipoproteins in human iPSC-derived neurons with familial Alzheimer's disease mutations. *Cell Rep*. 2016;17(3):759–773.
4. Siegel G, et al. The Alzheimer's disease γ-secretase generates higher 42:40 ratios for β-amyloid than for p3 peptides. *Cell Rep*. 2017;19(10):1967–1976.
5. Arber C, et al. Familial Alzheimer's disease patient-derived neurons reveal distinct mutation-specific effects on amyloid beta. *Mol Psychiatry*. 2020;25(11):2919–2931.
6. Kwart D, et al. A large panel of isogenic APP and PSEN1 mutant human iPSC neurons reveals shared endosomal abnormalities mediated by APP beta-CTFs, not abeta. *Neuron*. 2019;104(2):256–270.e255.
7. Tracy TE, et al. Tau interactome maps synaptic and mitochondrial processes associated with neurodegeneration. *Cell*. 2022;185(4):712–728.e714.
8. Lomoio S, et al. 3D bioengineered neural tissue generated from patient-derived iPSCs mimics time-dependent phenotypes and transcriptional features of Alzheimer's disease. *Mol Psychiatry*. 2023;28(12):5390–5401.
9. Buchholz S, et al. The tau isoform 1N4R confers vulnerability of MAPT knockout human iPSC-derived neurons to amyloid beta and phosphorylated tau-induced neuronal dysfunction. *Alzheimers Dement*. 2025;21(5):e14403.
10. Labra SR, et al. Autophagy activators normalize aberrant Tau proteostasis and rescue synapses in human familial Alzheimer's disease iPSC-derived cortical organoids. *Adv Sci (Weinh)*. 2026;13(17):e14783.
11. Samelson AJ, et al. CRISPR screens in iPSC-derived neurons reveal principles of tau proteostasis. *Cell*. 2026;189(5):1517–1534.e1519.
12. Guttikonda SR, et al. Fully defined human pluripotent stem cell-derived microglia and tri-culture system model C3 production in Alzheimer's disease. *Nat Neurosci*. 2021;24(3):343–354.
13. Hung C, et al. Mislocalization of nucleic acids is a convergent and targetable mechanism in Alzheimer's disease and frontotemporal dementia. *Cell Rep*. 2025;44(7):115867.
14. Arber C, et al. Familial Alzheimer's disease mutations in PSEN1 lead to premature human stem cell neurogenesis. *Cell Rep*. 2021;34(2):108615.
15. Arber C, et al. The presenilin 1 mutation P436S causes familial Alzheimer's disease with elevated Aβ43 and atypical clinical manifestations. *Alzheimers Dement*. 2024;20(7):4717–4726.
16. Ziff OJ, et al. Mutations in PSEN1 predispose inflammation in an astrocyte model of familial Alzheimer's disease through disrupted regulated intramembrane proteolysis. *Mol Neurodegener*. 2025;20(1):73.
17. Moreno CL, et al. iPSC-derived familial Alzheimer's PSEN2 (N141I) cholinergic neurons exhibit mutation-dependent molecular pathology corrected by insulin signaling. *Mol Neurodegener*. 2018;13(1):33.
18. MacMullen C, et al. Mitochondrial dynamics and bioenergetics in Alzheimer's induced pluripotent stem cell-derived neurons. *Brain*. 2025;148(4):1405–1420.
19. Shi Y, et al. A human stem cell model of early Alzheimer's disease pathology in Down syndrome. *Sci Transl Med*. 2012;4(124):124ra129.
20. Gonzalez C, et al. Modeling amyloid beta and tau pathology in human cerebral organoids. *Mol Psychiatry*. 2018;23(12):2363–2374.
21. Alić I, et al. Patient-specific Alzheimer-like pathology in trisomy 21 cerebral organoids reveals BACE2 as a gene dose-sensitive AD suppressor in human brain. *Mol Psychiatry*. 2021;26(10):5766–5788.
22. Wu CI, et al. APP and DYRK1A regulate axonal and synaptic vesicle protein networks and mediate Alzheimer's pathology in trisomy 21 neurons. *Mol Psychiatry*. 2022;27(4):1970–1989.
23. Jin M, et al. Type-I-interferon signaling drives microglial dysfunction and senescence in human iPSC models of Down syndrome and Alzheimer's disease. *Cell Stem Cell*. 2022;29(7):1135–1153.e1138.
24. Curtis ME, et al. Dysfunction of the retromer complex system contributes to amyloid and tau pathology in a stem cell model of Down syndrome. *Alzheimers Dement (N Y)*. 2022;8(1):e12334.
25. Fertan E, et al. Cerebral organoids with chromosome 21 trisomy secrete Alzheimer's disease-related soluble aggregates detectable by single-molecule-fluorescence and super-resolution microscopy. *Mol Psychiatry*. 2024;29(2):369–386.

26. Wang C, et al. Gain of toxic apolipoprotein E4 effects in human iPSC-derived neurons is ameliorated by a small-molecule structure corrector. *Nature medicine*. 2018;24(5):647–657.
27. Lin YT, et al. *APOE4* causes widespread molecular and cellular alterations associated with Alzheimer's disease phenotypes in human iPSC-derived brain cell types. *Neuron*. 2018;98(6):1141–1154.e1147.
28. Meyer K, et al. REST and neural gene network dysregulation in iPSC models of Alzheimer's disease. *Cell Rep*. 2019;26(5):1112–1127.e1119.
29. Zhao J, et al. APOE4 exacerbates synapse loss and neurodegeneration in Alzheimer's disease patient iPSC-derived cerebral organoids. *Nat Commun*. 2020;11(1):5540.
30. Tcw J, et al. Cholesterol and matrisome pathways dysregulated in astrocytes and microglia. *Cell*. 2022;185(13):2213–2233.e2225.
31. Lee H, et al. ApoE4-dependent lysosomal cholesterol accumulation impairs mitochondrial homeostasis and oxidative phosphorylation in human astrocytes. *Cell Rep*. 2023;42(10):113183.
32. Rao A, et al. Microglia depletion reduces human neuronal APOE4-related pathologies in a chimeric Alzheimer's disease model. *Cell Stem Cell*. 2025;32(1):86–104.e107.
33. Lish AM, et al. Astrocyte induction of disease-associated microglia is suppressed by acute exposure to fAD neurons in human iPSC triple cultures. *Cell Rep*. 2025;44(6):115777.
34. Lee SI, et al. Astrocyte priming enhances microglial Aβ clearance and is compromised by APOE4. *Nat Commun*. 2025;16(1):7551.
35. Ramirez AM, et al. Ancestral genomic functional differences in oligodendroglia: Implications for Alzheimer's disease. *Alzheimers Dement*. 2025;21(9):e70593.
36. Nugent AA, et al. TREM2 regulates microglial cholesterol metabolism upon chronic phagocytic challenge. *Neuron*. 2020;105(5):837–854.e839.
37. McQuade A, et al. Gene expression and functional deficits underlie TREM2-knockout microglia responses in human models of Alzheimer's disease. *Nat Commun*. 2020;11(1):5370.
38. Knupp A, et al. Depletion of the AD risk gene SORL1 selectively impairs neuronal endosomal traffic independent of amyloidogenic APP processing. *Cell Rep*. 2020;31(9):107719.
39. Hung C, et al. SORL1 deficiency in human excitatory neurons causes APP-dependent defects in the endolysosome-autophagy network. *Cell Rep*. 2021;35(11):109259.
40. Juul-Madsen K, et al. Familial Alzheimer's disease mutation identifies novel role of SORLA in release of neurotrophic exosomes. *Alzheimers Dement*. 2025;21(9):e70591.
41. Bedford LM, et al. Alzheimer's disease-associated PLCG2 variants alter microglial state and function in human induced pluripotent stem cell-derived microglia-like cells. *Alzheimers Dement*. 2025;21(10):e70772.
42. Kawatani K, et al. ABCA7 deficiency causes neuronal dysregulation by altering mitochondrial lipid metabolism. *Mol Psychiatry*. 2024;29(3):809–819.
43. Pérez MJ, et al. Loss of function of the mitochondrial peptidase PITRM1 induces proteotoxic stress and Alzheimer's disease-like pathology in human cerebral organoids. *Mol Psychiatry*. 2021;26(10):5733–5750.
44. Lagomarsino VN, et al. Stem cell-derived neurons reflect features of protein networks, neuropathology, and cognitive outcome of their aged human donors. *Neuron*. 2021;21:00578.
45. Chen X, et al. Modeling sporadic Alzheimer's disease in human brain organoids under serum exposure. *Adv Sci (Weinh)*. 2021;8(18):e2101462.
46. Ji Y, et al. Alzheimer's disease patient brain extracts induce multiple pathologies in novel vascularized neuroimmune organoids for disease modeling and drug discovery. *Mol Psychiatry*. 2025;30(10):4558–4575.
47. Devine MJ, et al. Parkinson's disease induced pluripotent stem cells with triplication of the α-synuclein locus. *Nat Commun*. 2011;2:440.
48. Byers B, et al. SNCA triplication Parkinson's patient's iPSC-derived DA neurons accumulate α-synuclein and are susceptible to oxidative stress. *PloS one*. 2011;6(11):e26159.
49. Ludtmann MHR, et al. α-synuclein oligomers interact with ATP synthase and open the permeability transition pore in Parkinson's disease. *Nat Commun*. 2018;9(1):2293.
50. Burbidge K, et al. LGALS3 (galectin 3) mediates an unconventional secretion of SNCA/α-synuclein in response to lysosomal membrane damage by the autophagic-lysosomal pathway in human midbrain dopamine neurons. *Autophagy*. 2022;18(5):1020–1048.
51. Iannielli A, et al. Modeling native and seeded Synuclein aggregation and related cellular dysfunctions in dopaminergic neurons derived by a new set of isogenic iPSC lines with SNCA multiplications. *Cell Death Dis*. 2022;13(10):881.

52. Virdi GS, et al. Protein aggregation and calcium dysregulation are hallmarks of familial Parkinson's disease in midbrain dopaminergic neurons. *NPJ Parkinsons Dis*. 2022;8(1):162.
53. Patikas N, et al. Single-cell transcriptomics identifies perturbed molecular pathways in midbrain organoids using α-synuclein triplication Parkinson's disease patient-derived iPSCs. *Neurosci Res*. 2023;195:13–28.
54. Drobny A, et al. Reciprocal effects of alpha-synuclein aggregation and lysosomal homeostasis in synucleinopathy models. *Transl Neurodegener*. 2023;12(1):31.
55. Kim MS, et al. Advanced human iPSC-based preclinical model for Parkinson's disease with optogenetic alpha-synuclein aggregation. *Cell Stem Cell*. 2023;30(7):973–986.e911.
56. White AJ, et al. A stem cell-based assay platform demonstrates alpha-synuclein dependent synaptic dysfunction in patient-derived cortical neurons. *NPJ Parkinsons Dis*. 2024;10(1):107.
57. Chedid J, et al. Dopamine and cortical neurons with different Parkinsonian mutations show variation in lysosomal and mitochondrial dysfunction. *NPJ Parkinsons Dis*. 2025;11(1):177.
58. Balta D, et al. Recombinant cathepsins B and L promote α-synuclein clearance and restore lysosomal function in human and murine models with α-synuclein pathology. *Mol Neurodegener*. 2025;20(1):95.
59. Statoulla E, et al. SNCA triplication disrupts proteostasis and extracellular architecture prior to neurodegeneration in human midbrain organoids. *NPJ Parkinsons Dis*. 2026;12(1):81.
60. Nguyen HN, et al. LRRK2 mutant iPSC-derived DA neurons demonstrate increased susceptibility to oxidative stress. *Cell Stem Cell*. 2011;8(3):267–280.
61. Sánchez-Danés A, et al. Disease-specific phenotypes in dopamine neurons from human iPS-based models of genetic and sporadic Parkinson's disease. *EMBO Mol Med*. 2012;4(5):380–395.
62. Fernández-Santiago R, et al. Aberrant epigenome in iPSC-derived dopaminergic neurons from Parkinson's disease patients. *EMBO Mol Med*. 2015;7(12):1529–1546.
63. Hsieh CH, et al. Functional impairment in miro degradation and mitophagy is a shared feature in familial and sporadic Parkinson's disease. *Cell Stem Cell*. 2016;19(6):709–724.
64. Schwab AJ, et al. Decreased sirtuin deacetylase activity in LRRK2 G2019S iPSC-derived dopaminergic neurons. *Stem Cell Reports*. 2017;9(6):1839–1852.
65. Schöndorf DC, et al. The NAD+ precursor nicotinamide riboside rescues mitochondrial defects and neuronal loss in iPSC and fly models of Parkinson's sisease. *Cell Rep*. 2018;23(10):2976–2988.
66. Kim JW, et al. Defects in mRNA translation in LRRK2-mutant hiPSC-derived dopaminergic neurons lead to dysregulated calcium homeostasis. *Cell Stem Cell*. 2020;27(4):633–645.e637.
67. Panagiotakopoulou V, et al. Interferon-γ signaling synergizes with LRRK2 in neurons and microglia derived from human induced pluripotent stem cells. *Nat Commun*. 2020;11(1):5163.
68. Ramos-Gonzalez P, et al. Astrocytic atrophy as a pathological feature of Parkinson's disease with LRRK2 mutation. *NPJ Parkinsons Dis*. 2021;7(1):31.
69. Carola G, et al. Parkinson's disease patient-specific neuronal networks carrying the LRRK2 G2019S mutation unveil early functional alterations that predate neurodegeneration. *NPJ Parkinsons Dis*. 2021;7(1):55.
70. Kedariti M, et al. LRRK2 kinase activity regulates GCase level and enzymatic activity differently depending on cell type in Parkinson's disease. *NPJ Parkinsons Dis*. 2022;8(1):92.
71. MacDougall EJ, et al. LRRK2 kinase mediates increased GCase activity in microglia in response to IFNγ-induced proinflammatory stimulation. *NPJ Parkinsons Dis*. 2026;12(1):99.
72. Kim BW, et al. Human motor neurons with SOD1-G93A mutation generated from CRISPR/Cas9 gene-edited iPSCs develop pathological features of amyotrophic lateral sclerosis. *Front Cell Neurosci*. 2020;14:604171.
73. Chung SY, et al. Parkin and PINK1 patient iPSC-derived midbrain dopamine neurons exhibit mitochondrial dysfunction and α-synuclein accumulation. *Stem Cell Reports*. 2016;7(4):664–677.
74. Kano M, et al. Reduced astrocytic reactivity in human brains and midbrain organoids with PRKN mutations. *NPJ Parkinsons Dis*. 2020;6(1):33.
75. Peng W, et al. Parkin regulates amino acid homeostasis at mitochondria-lysosome (M/L) contact sites in Parkinson's disease. *Sci Adv*. 2023;9(29):eadh3347.
76. Morrone Parfitt G, et al. Disruption of lysosomal proteolysis in astrocytes facilitates midbrain organoid proteostasis failure in an early-onset Parkinson's disease model. *Nat Commun*. 2024;15(1):447.
77. Weiss F, et al. Astrocytes contribute to toll-like receptor 2-mediated neurodegeneration and alpha-synuclein pathology in a human midbrain Parkinson's model. *Transl Neurodegener*. 2024;13(1):62.
78. Tripathi U, et al. Upregulated ECM genes and increased synaptic activity in Parkinson's human DA neurons with PINK1/ PRKN mutations. *NPJ Parkinsons Dis*. 2024;10(1):103.

79. Schöndorf DC, et al. iPSC-derived neurons from GBA1-associated Parkinson's disease patients show autophagic defects and impaired calcium homeostasis. *Nat Commun*. 2014;5:4028.
80. Aflaki E, et al. A new glucocerebrosidase chaperone reduces α-synuclein and glycolipid levels in iPSC-derived dopaminergic neurons from patients with Gaucher disease and Parkinsonism. *J Neurosci*. 2016;36(28):7441–7452.
81. Burbulla LF, et al. A modulator of wild-type glucocerebrosidase improves pathogenic phenotypes in dopaminergic neuronal models of Parkinson's disease. *SciTransl Med*. 2019;11(514):eaau6870.
82. Rosety I, et al. Impaired neuron differentiation in GBA-associated Parkinson's disease is linked to cell cycle defects in organoids. *NPJ Parkinsons Dis*. 2023;9(1):166.
83. Jacquemyn J, et al. Glucosylceramide-induced ectosomes propagate pathogenic α-synuclein in Parkinson's disease. *Nat Cell Biol*. 2026;28(3):492–506.
84. Lin L, et al. Molecular features underlying neurodegeneration identified through *in vitro* modeling of genetically diverse Parkinson's disease patients. *Cell Rep*. 2016;15(11):2411–2426.
85. Manders L, et al. VPS35 mutation inhibits PINK1/parkin-mediated mitophagy via increased LRRK2 kinase activity. *Brain*. 2025:awaf414.
86. Chemla A, et al. Parkinson's disease mutant Miro1 causes mitochondrial dysfunction and dopaminergic neuron loss. *Brain*. 2025;148(10):3607–3622.
87. Laperle AH, et al. iPSC modeling of young-onset Parkinson's disease reveals a molecular signature of disease and novel therapeutic candidates. *Nature medicine*. 2020;26(2):289–299.
88. Almeida S, et al. Modeling key pathological features of frontotemporal dementia with C9ORF72 repeat expansion in iPSC-derived human neurons. *Acta Neuropathol*. 2013;126(3):385–399.
89. Bursch F, et al. Altered calcium dynamics and glutamate receptor properties in iPSC-derived motor neurons from ALS patients with C9orf72, FUS, SOD1 or TDP43 mutations. *Hum Mol Genet*. 2019;28(17):2835–2850.
90. Coyne AN, et al. Nuclear accumulation of CHMP7 initiates nuclear pore complex injury and subsequent TDP-43 dysfunction in sporadic and familial ALS. *Sci Transl Med*. 2021;13(604):eabe1923.
91. Workman MJ, et al. Large-scale differentiation of iPSC-derived motor neurons from ALS and control subjects. *Neuron*. 2023;111(8):1191–1204.e1195.
92. Westergard T, et al. Cell-to-cell transmission of dipeptide repeat proteins linked to C9orf72-ALS/FTD. *Cell Rep*. 2016;17(3):645–652.
93. Perkins EM, et al. Altered network properties in C9ORF72 repeat expansion cortical neurons are due to synaptic dysfunction. *Mol Neurodegener*. 2021;16(1):13.
94. Szebényi K, et al. Human ALS/FTD brain organoid slice cultures display distinct early astrocyte and targetable neuronal pathology. *Nat Neurosci*. 2021;24(11):1542–1554.
95. Guo W, et al. CRISPR/Cas9 screen in human iPSC-derived cortical neurons identifies NEK6 as a novel disease modifier of C9orf72 poly(PR) toxicity. *Alzheimers Dement*. 2023;19(4):1245–1259.
96. Banerjee P, et al. Cell-autonomous immune dysfunction driven by disrupted autophagy in C9orf72-ALS iPSC-derived microglia contributes to neurodegeneration. *Sci Adv*. 2023;9(16):eabq0651.
97. Vahsen BF, et al. C9orf72-ALS human iPSC microglia are pro-inflammatory and toxic to co-cultured motor neurons via MMP9. *Nat Commun*. 2023;14(1):5898.
98. Harley P, et al. Aberrant axon initial segment plasticity and intrinsic excitability of ALS hiPSC motor neurons. *Cell Rep*. 2023;42(12):113509.
99. Beckers J, Van Damme P. Toxic gain-of-function mechanisms in C9orf72 ALS-FTD neurons drive autophagy and lysosome dysfunction. *Autophagy*. 2024;20(9):2102–2104.
100. Ljubikj T, et al. PU.1 restores microglial dysfunction caused by C9ORF72 repeat expansions in neural organoids. *Brain*. 2026;149(3):801–817.
101. Chen H, et al. Modeling ALS with iPSCs reveals that mutant SOD1 misregulates neurofilament balance in motor neurons. *Cell Stem Cell*. 2014;14(6):796–809.
102. Luisier R, et al. Intron retention and nuclear loss of SFPQ are molecular hallmarks of ALS. *Nat Commun*. 2018;9(1):2010.
103. Granatiero V, et al. Modulation of the IGF1R-MTOR pathway attenuates motor neuron toxicity of human ALS SOD1(G93A) astrocytes. *Autophagy*. 2021;17(12):4029–4042.
104. Pereira JD, et al. Human sensorimotor organoids derived from healthy and amyotrophic lateral sclerosis stem cells form neuromuscular junctions. *Nat Commun*. 2021;12(1):4744.
105. Namboori SC, et al. Single-cell transcriptomics identifies master regulators of neurodegeneration in SOD1 ALS iPSC-derived motor neurons. *Stem Cell Reports*. 2021;16(12):3020–3035.

106. Tsioras K, et al. Analysis of proteome-wide degradation dynamics in ALS SOD1 iPSC-derived patient neurons reveals disrupted VCP homeostasis. *Cell Rep*. 2023;42(10):113160.
107. Ho R, et al. ALS disrupts spinal motor neuron maturation and aging pathways within gene co-expression networks. *Nat Neurosci*. 2016;19(9):1256–1267.
108. Yu CH, et al. TDP-43 triggers mitochondrial DNA release via mPTP to activate cGAS/STING in ALS. *Cell*. 2020;183(3):636–649.e618.
109. Melamed Z, et al. Premature polyadenylation-mediated loss of stathmin-2 is a hallmark of TDP-43-dependent neurodegeneration. *Nat Neurosci*. 2019;22(2):180–190.
110. Smethurst P, et al. Distinct responses of neurons and astrocytes to TDP-43 proteinopathy in amyotrophic lateral sclerosis. *Brain*. 2020;143(2):430–440.
111. Ratti A, et al. Chronic stress induces formation of stress granules and pathological TDP-43 aggregates in human ALS fibroblasts and iPSC-motoneurons. *Neurobiol Dis*. 2020;145:105051.
112. Fazal R, et al. HDAC6 inhibition restores TDP-43 pathology and axonal transport defects in human motor neurons with TARDBP mutations. *EMBO J*. 2021;40(7):e106177.
113. Altman T, et al. Axonal TDP-43 condensates drive neuromuscular junction disruption through inhibition of local synthesis of nuclear encoded mitochondrial proteins. *Nat Commun*. 2021;12(1):6914.
114. Ma XR, et al. TDP-43 represses cryptic exon inclusion in the FTD-ALS gene UNC13A. *Nature*. 2022;603(7899):124–130.
115. Gleixner AM, et al. NUP62 localizes to ALS/FTLD pathological assemblies and contributes to TDP-43 insolubility. *Nat Commun*. 2022;13(1):3380.
116. Rojas F, et al. Mature iPSC-derived astrocytes of an ALS/FTD patient carrying the TDP43(A90V) mutation display a mild reactive state and release polyP toxic to motoneurons. *Front Cell Dev Biol*. 2023;11:1226604.
117. Lépine S, et al. Homozygous ALS-linked mutations in TARDBP/TDP-43 lead to hypoactivity and synaptic abnormalities in human iPSC-derived motor neurons. *iScience*. 2024;27(3):109166.
118. Vandoorne T, et al. Differentiation but not ALS mutations in FUS rewires motor neuron metabolism. *Nat Commun*. 2019;10(1):4147.
119. Castillo Bautista CM, et al. Obatoclax rescues FUS-ALS phenotypes in iPSC-derived neurons by inducing autophagy. *Cells*. 2023;12(18):2247.
120. Guo W, et al. HDAC6 inhibition reverses axonal transport defects in motor neurons derived from FUS-ALS patients. *Nat Commun*. 2017;8(1):861.
121. Stoklund Dittlau K, et al. FUS-ALS hiPSC-derived astrocytes impair human motor units through both gain-of-toxicity and loss-of-support mechanisms. *Mol Neurodegener*. 2023;18(1):5.
122. Szewczyk B, et al. FUS ALS neurons activate major stress pathways and reduce translation as an early protective mechanism against neurodegeneration. *Cell Rep*. 2023;42(2):112025.
123. Otomo A, et al. Investigation of early axonal phenotypes in an iPSC-derived ALS cellular model using a microfluidic device. *Front Cell Neurosci*. 2025;19:1590732.
124. Rafiee MR, et al. RNA polymerase II-associated proteins reveal pathways affected in VCP-related amyotrophic lateral sclerosis. *Brain*. 2023;146(6):2547–2556.
125. Dubey SK, et al. Aberrant nuclear pore complex degradation contributes to neurodegeneration in VCP disease. *Neuron*. 2026;114(5):850–867.e858.
126. Hall CE, et al. Progressive motor neuron pathology and the role of astrocytes in a human stem cell model of VCP-related ALS. *Cell Rep*. 2017;19(9):1739–1749.
127. Clarke BE, et al. Human VCP mutant ALS/FTD microglia display immune and lysosomal phenotypes independently of GPNMB. *Mol Neurodegener*. 2024;19(1):90.
128. Hung C, Patani R. Elevated 4R tau contributes to endolysosomal dysfunction and neurodegeneration in VCP-related frontotemporal dementia. *Brain*. 2024;147(3):970–979.
129. Funes S, et al. Expression of ALS-PFN1 impairs vesicular degradation in iPSC-derived microglia. *Nat Commun*. 2024;15(1):2497.
130. Vieira de Sá R, et al. ATAXIN-2 intermediate-length polyglutamine expansions elicit ALS-associated metabolic and immune phenotypes. *Nat Commun*. 2024;15(1):7484.
131. Fujimori K, et al. Modeling sporadic ALS in iPSC-derived motor neurons identifies a potential therapeutic agent. *Nature medicine*. 2018;24(10):1579–1589.
132. Huber N, et al. Frontotemporal dementia patient-derived iPSC neurons show cell pathological hallmarks and evidence for synaptic dysfunction and DNA damage. *Mol Psychiatry*. 2026;31(3):1500–1516.
133. Bowles KR, et al. ELAVL4, splicing, and glutamatergic dysfunction precede neuron loss in MAPT mutation cerebral organoids. *Cell*. 2021;184(17):4547–4563.e4517.

134. Sohn PD, et al. Pathogenic tau impairs axon initial segment plasticity and excitability homeostasis. *Neuron*. 2019;104(3):458–470.
135. Paonessa F, et al. Microtubules deform the nuclear membrane and disrupt nucleocytoplasmic transport in tau-mediated frontotemporal dementia. *Cell Rep*. 2019;26(3):582–593.e585.
136. Sposito T, et al. Developmental regulation of tau splicing is disrupted in stem cell-derived neurons from frontotemporal dementia patients with the 10 + 16 splice-site mutation in MAPT. *Hum Mol Genet*. 2015;24(18):5260–5269.
137. Ehrlich M, et al. Distinct neurodegenerative changes in an induced pluripotent stem cell model of frontotemporal dementia linked to mutant TAU protein. *Stem Cell Reports*. 2015;5(1):83–96.
138. Biswas MHU, et al. MMP-9 and MMP-2 contribute to neuronal cell death in iPSC models of frontotemporal dementia with MAPT mutations. *Stem Cell Reports*. 2016;7(3):316–324.
139. Verheyen A, et al. Genetically engineered iPSC-derived FTDP-17 MAPT neurons display mutation-specific neurodegenerative and neurodevelopmental phenotypes. *Stem Cell Reports*. 2018;11(2):363–379.
140. Esteras N, et al. Mitochondrial ROS control neuronal excitability and cell fate in frontotemporal dementia. *Alzheimers Dement*. 2022;18(2):318–338.
141. Parra Bravo C, et al. Human iPSC 4R tauopathy model uncovers modifiers of tau propagation. *Cell*. 2024;187(10):2446–2464.e2422.
142. Iyer AK, et al. Cell autonomous microglia defects in a stem cell model of frontotemporal dementia tau. *Mol Psychiatry*. 2025;30(10):4776–4791.
143. Logan T, et al. Rescue of a lysosomal storage disorder caused by Grn loss of function with a brain penetrant progranulin biologic. *Cell*. 2021;184(18):4651–4668.e4625.
144. Bossolasco P, et al. GRN-/- iPSC-derived cortical neurons recapitulate the pathological findings of both frontotemporal lobar degeneration and neuronal ceroidolipofuscinosis. *Neurobiol Dis*. 2022;175:105891.
145. de Majo M, et al. Granulin loss of function in human mature brain organoids implicates astrocytes in TDP-43 pathology. *Stem Cell Reports*. 2023;18(3):706–719.
146. Hasan S, et al. Multi-modal proteomic characterization of lysosomal function and proteostasis in progranulin-deficient neurons. *Mol Neurodegener*. 2023;18(1):87.
147. Hu W, et al. Direct conversion of normal and Alzheimer's disease human fibroblasts into neuronal cells by small molecules. *Cell Stem Cell*. 2015;17(2):204–212.
148. Mertens J, et al. Directly reprogrammed human neurons retain aging-associated transcriptomic signatures and reveal age-related nucleocytoplasmic defects. *Cell Stem Cell*. 2015;17(6):705–718.
149. Mertens J, et al. Age-dependent instability of mature neuronal fate in induced neurons from Alzheimer's patients. *Cell Stem Cell*. 2021;28(9):1533–1548.e1536.
150. Herdy JR, et al. Increased post-mitotic senescence in aged human neurons is a pathological feature of Alzheimer's disease. *Cell Stem Cell*. 2022;29(12):1637–1652.e1636.
151. Chou CC, et al. Proteostasis and lysosomal repair deficits in transdifferentiated neurons of Alzheimer's disease. *Nat Cell Biol*. 2025;27(4):619–632.
152. Caiazzo M, et al. Direct generation of functional dopaminergic neurons from mouse and human fibroblasts. *Nature*. 2011;476(7359):224–227.
153. Liu X, et al. Direct reprogramming of human fibroblasts into dopaminergic neuron-like cells. *Cell Res*. 2012;22(2):321–332.
154. Pfisterer U, et al. Direct conversion of human fibroblasts to dopaminergic neurons. *Proc Natl Acad Sci U S A*. 2011;108(25):10343–11048.
155. Liu ML, et al. Direct lineage reprogramming reveals disease-specific phenotypes of motor neurons from human ALS patients. *Cell Rep*. 2016;14(1):115–128.
156. Woo E, et al. Investigation of mitochondrial phenotypes in motor neurons derived by direct conversion of fibroblasts from familial ALS subjects. *bioRxiv*. 2025:2025.2002.2013.637962 [Preprint]. February 637917, 632025. Available from: https://doi.org/637910.631101/632025.637902.637913.637962
157. Capano LS, et al. Recapitulation of endogenous 4R tau expression and formation of insoluble tau in directly reprogrammed human neurons. *Cell Stem Cell*. 2022;29(6):918–932.e918.
158. Victor MB, et al. Striatal neurons directly converted from Huntington's disease patient fibroblasts recapitulate age-associated disease phenotypes. *Nat Neurosci*. 2018;21(3):341–352.
159. Kraskovskaya NA, et al. Direct reprogramming of somatic skin cells from a patient with Huntington's disease into striatal neurons to create models of pathology. *Dokl Biol Sci*. 2024;515(1):15–19.
